# Fifty Years Later, and We Still Don't Know About Badges of Status

**DOI:** 10.1002/ece3.73578

**Published:** 2026-04-29

**Authors:** Alfredo Sánchez‐Tójar, Pietro B. D'Amelio

**Affiliations:** ^1^ Department of Evolutionary Biology Bielefeld University Bielefeld Germany; ^2^ CNC‐UC, Center for Neuroscience and Cell Biology University of Coimbra Coimbra Portugal; ^3^ CIBB, Center for Innovative Biomedicine and Biotechnology University of Coimbra Coimbra Portugal; ^4^ Department of Biology Reed College Portland Oregon USA

**Keywords:** aggression, carotenoids, condition‐dependent traits, dominance hierarchy, melanin, sexual selection, status signaling hypothesis

## Abstract

A recent meta‐analysis concluded that the badge of status hypothesis is the most parsimonious explanation for an observed association between measures of aggression and the degree or area of coloration across animals. We argue that the premise for the conclusion that aggression and coloration are positively associated relies on a biased, largely incomplete and heterogeneous representation of the available evidence. In addition, we identified severe problems with the calculation and interpretation of the correlations and the heterogeneity, as well as with data extraction and the publication bias tests. Our re‐analyses challenge the original conclusions. Once heterogeneity—which, in contrast to the original study, our re‐analysis shows to be high—and publication bias are properly assessed and accounted for, we find low generality of the relationship between aggression and coloration across animals. In addition, our analyses suggest that evidence changes dramatically depending on the source of the effect size, with evidence obtained directly from correlations and mean group comparisons being statistically nonsignificant and largely different from evidence obtained by transforming inferential statistics (*t*, *F*, and *χ* ^2^), which raises strong methodological concerns. Furthermore, by re‐extracting 11 out of the 74 studies included in the original meta‐analysis (15%), we found errors and inconsistencies that raise further concerns about the validity of the full dataset. We give directions to guide future meta‐analytic efforts in this and other topics, showcasing common misunderstandings in systematic reviews and meta‐analyses, including effect size calculation and transformation. We conclude that after 50 years since its conception, the jury is still out regarding the validity and generality of the badge of status hypothesis to explain individual differences in coloration traits across animals.

## Graphical abstract

Overall, aggression and coloration are positively associated across species, but the generality of this relationship is low, the relationship depends on effect size origin, and the dataset requires further verification, which raises methodological concerns.

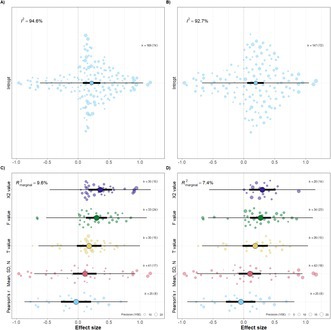

## 1 Introduction

Animals' intraspecific color variation may be adaptive (Cuthill et al. 2017). The intensity, size or hue of color patches may positively correlate with individual fighting abilities and therefore function as a signal during agonistic interactions (Rohwer 1975; Senar 2006). Ritualization of agonistic interactions is often selected for, since both parties can spare the costs of the contest (Berglund et al. 1996; Emlen 2008). For the communication of fighting abilities to be reliable, the sender's signal is expected to be predominantly honest (Dashtbali et al. 2024; Moore et al. 2022). As a consequence, we expect a correlation between the intensity, size or hue of color patches and fitness costs, either through trade‐offs or because such signals are tied to vital processes (Hill et al. 2023). The explanation of the origin of the costs of color patches and the possibility of cheating has sparked a lot of fruitful experimental and theoretical research (e.g., Számadó et al. 2023; Tibbetts and Dale 2004; Weaver et al. 2017). In this context, finding general patterns of association between animal color and aggressive behavior is an important endeavor. Ruckman et al. (2024) used a meta‐analytic approach to synthesize literature on this topic and explored three hypotheses: (1) aggression and color are positively correlated because of a genetic link between them (“melanocortin pleiotropy hypothesis”; Roulin 2016); (2) color signal is condition‐dependent (“condition‐dependent hypothesis”; Hill 2011); and (3) colors can be signals or “badges” of (social) status (“badge of status hypothesis”, AKA “status signaling hypothesis”; Rohwer 1975). Ruckman et al. (2024) argued that, depending on the type of pigmentation (i.e., eumelanic, carotenoid, or structural), there are different expectations for the three hypotheses. Specifically, they reasoned that the cost of producing melanin versus carotenoid is likely different because, while probably negligible for melanin (Roulin 2016), it is considered substantial for carotenoids, which are diet‐dependent (Hill et al. 2023; Svensson and Wong 2011). Therefore, finding an association between aggression and coloration only for melanin patches would support the *melanocortin pleiotropy hypothesis*, whereas finding the association only for carotenoids would favor the *condition‐dependent hypothesis*. Ruckman et al. (2024) found a positive association between color (patch size and/or darkness) and aggression independently of pigment type and concluded that the most likely widespread mechanism among animals is the *badge of status hypothesis* because this hypothesis is agnostic to the type of pigment.

In this Reply, we challenge this interpretation by identifying several biases, errors and misunderstandings in Ruckman et al. (2024). First, a simple comparison of Ruckman et al. (2024)'s study selection to a meta‐analysis testing the *badge of status hypothesis* in one bird species (Sánchez‐Tójar et al. 2018) showcases how Ruckman et al. (2024)'s search likely missed numerous relevant studies, biasing the sampling. Second, we corrected a code mistake that affected the calculation of 25% of all effect sizes in the original analysis, as well as highlighted that the Fisher Z‐transformation should not have been performed on this dataset (Jacobs and Viechtbauer 2017). We re‐analyzed the data accordingly and show how the presence of analytical errors in the estimation and interpretation of heterogeneity and publication bias (small‐study effects) lead to less univocal results, prompting us to propose a different interpretation. Third, we compared results (i.e., overall effects) between the different types of statistics used (i.e., the origin of the effect sizes) to highlight possible undetected biases in the original effect size calculation (e.g., in the direction of effect sizes). Fourth, we subsequently performed a data extraction verification on 15% of the included studies across different types of statistics used, which confirmed errors in data extraction and further raised concerns about the original dataset. Correcting these errors further weakened the association between aggression and coloration, showing a lack of robustness of the overall evidence. We conclude by outlining best practices for avoiding similar errors in future systematic reviews and meta‐analyses.

## 2 Methods

### 2.1 Literature Sampling

Using the keyword search string provided by Ruckman et al. (2024), we performed a search in Scopus to compare the studies present in the original search of Ruckman et al. (2024) before any exclusion (*N* = 731 records) to the published studies included in the meta‐analysis of Sánchez‐Tójar et al. (2018; *N* = 13) as a proof‐of‐concept test of the comprehensiveness of Ruckman et al. (2024)'s search strategy.

### 2.2 Re‐Calculation of Effect Sizes of Biserial Correlations

By exploring the data and scripts provided by Ruckman et al. (2024) on a GitHub repository (https://github.com/sruckman/meta‐analysis), we noticed that the custom function used to calculate biserial correlations (*r*_bis_) missed a set of parentheses (represented by “[]” in Equation 1) while calculating parameter “*d*” (i.e., the effect size of interest). Here, we provide a fixed version of this function:

(1) $$r_{\text{bis.fixed}}=\frac{\left({\bar{x}}_{D}-{\bar{x}}_{L}\right)}{\sqrt{\left[\left(\left(n_{L}-1\right)*{\mathrm{SD}}_{L}^{2}\right)+\left(\left(n_{D}-1\right)*{\mathrm{SD}}_{D}^{2}\right)\right]/\left(n_{L}+n_{D}-2\right)}},$$

where *x̄* refers to the mean of the dark (D) or light (L) group, *n* is the number of individuals in each group, and SD is the standard deviation. To explore differences between the original and the corrected dataset, we compare *r*_bis.original_ and *r*_bis.fixed_ for the 42 effect sizes (25% of the dataset) calculated as *r*_bis_ by Ruckman et al. (2024). In addition, to check the stability of the results, we simulated a larger dataset using the original group mean and SD values (D: 2.40 ± 0.80 and L: 2.25 ± 0.75). The simulation was performed using the R function “rnorm(),” and the sample size was fixed to 10 for each group, which coincides with the 1st quartile in Ruckman et al. (2024). We also ran the simulation using 70 as the sample size for each group, which corresponds to the maximum value in Ruckman et al. (2024). We present the results of this simulation in script “002_effect_size_simulation.R” (Sánchez‐Tójar and D'Amelio 2026), but shortly, *r*_bis.original_ (mean = 0.030, SD = 0.096) resulted in a lower average and variance than *r*_bis.fixed_ (mean = 0.085, SD = 0.273).

### 2.3 Re‐Analysis of Non‐Transformed Effect Sizes (*r* and *r*_*bis*_): Correct Approach

Beside the mistake in *r*_bis_ calculation, we found other shortcomings in Ruckman et al. (2024)'s effect size calculation procedure, so before re‐analyzing the data, we corrected each passage. First, contrary to the approach followed by Ruckman et al. (2024), Fisher Z‐transformation, which is often applied to Pearson's correlation coefficients (*r*), should not be performed when a meta‐analysis combines both *r* and *r*_bis_ correlations, because these require different transformations that cannot be directly compared (details in Jacobs and Viechtbauer 2017). Therefore, we used *r*, here referring to both *r* and *r*_bis_, as the effect size for our re‐analyses. Second, we used the broadly used function “escalc()” from the R package “metafor” v.4.6‐0 (Viechtbauer 2010) to calculate *r*_bis_ from means, SDs, and sample sizes, as well as to transform all *t* values present in the dataset into *r*. Third, we transformed all *χ* ^2^ values into Cramer's V (*V*), which is a measure of the association between two nominal variables from contingency tables of, importantly, different sizes (Cohen 2013; equation from Jané et al. 2024). Fourth and final change, we transformed all *F* values to eta‐squared, which allowed us to calculate the corresponding size of the omnibus *F* values (Ben‐Shachar et al. 2020; equation from Jané et al. 2024). The reason for using these alternative transformations was to be able to include all the data extracted by Ruckman et al. (2024), even those from relatively complex inferential models (e.g., *χ* ^2^ values from contingency tables larger than 2 × 2) without ignoring such complexity, while using approaches broadly accepted and implemented in established meta‐analytic R packages such as “metafor” (Viechtbauer 2010) and “effectsize” v.0.8.9 (Ben‐Shachar et al. 2020).

Once we corrected the effect size calculation, we fitted a phylogenetic multilevel intercept‐only model to explore the overall association between aggression and coloration. Then, we tested for small‐study effects by fitting a phylogenetic multilevel uni‐moderator meta‐regression including the square root of the inverse of the sample size as moderator (adjusted from equation 27 in Nakagawa et al. 2022).

### 2.4 Effect Size Origin

To test whether the calculated effect sizes differed depending on their origin (five levels: *r*, means‐SD‐n [*r*_bis_], *t* values, *F* values, *χ* ^2^ values), which could indicate potential methodological issues, we first explored the differences visually using the R package “ggstatsplot” v.0.12.5 (Patil 2021; Figure S2), and then ran a phylogenetic multilevel uni‐moderator meta‐regression with effect size origin as the moderator (more in Section 2.6).

### 2.5 Data Extraction Validation

The examination of effect size origin revealed potential issues with the originally extracted values. Consequently, we conducted a data extraction validation process for 15% of the studies (*N* = 11), following the inclusion/exclusion criteria reported by Ruckman et al. (2024: section “Assessment” in Figure 1 from Ruckman et al. 2024). We chose the studies pseudo‐randomly, ensuring that they would come from different effect size origins (i.e., *r*, means‐SD‐n [*r*_*bis*_], *t* values, *F* values, *χ* ^*2*^ values). For each revisited study, we extracted the reported statistical information for calculating all the possible effect sizes, assigned them and compared them with the ones extracted originally. We discussed each case and resolved any conflicts collectively. We provide the study‐by‐study, step‐by‐step detailed decision process as an .html document (see Supporting Information) generated with Quarto Markdown that combines narrative text written in markdown with executable code cells (Sánchez‐Tójar and D'Amelio 2026). We suggest that future meta‐analysts may want to use a similar template for transparently extracting data (see script “004_data_extraction_validation.qmd”; Sánchez‐Tójar and D'Amelio 2026). We updated the meta‐analytic dataset accordingly and re‐ran the main analysis, as well as the small‐study effects and effect size origin meta‐regressions.

**FIGURE 1:**
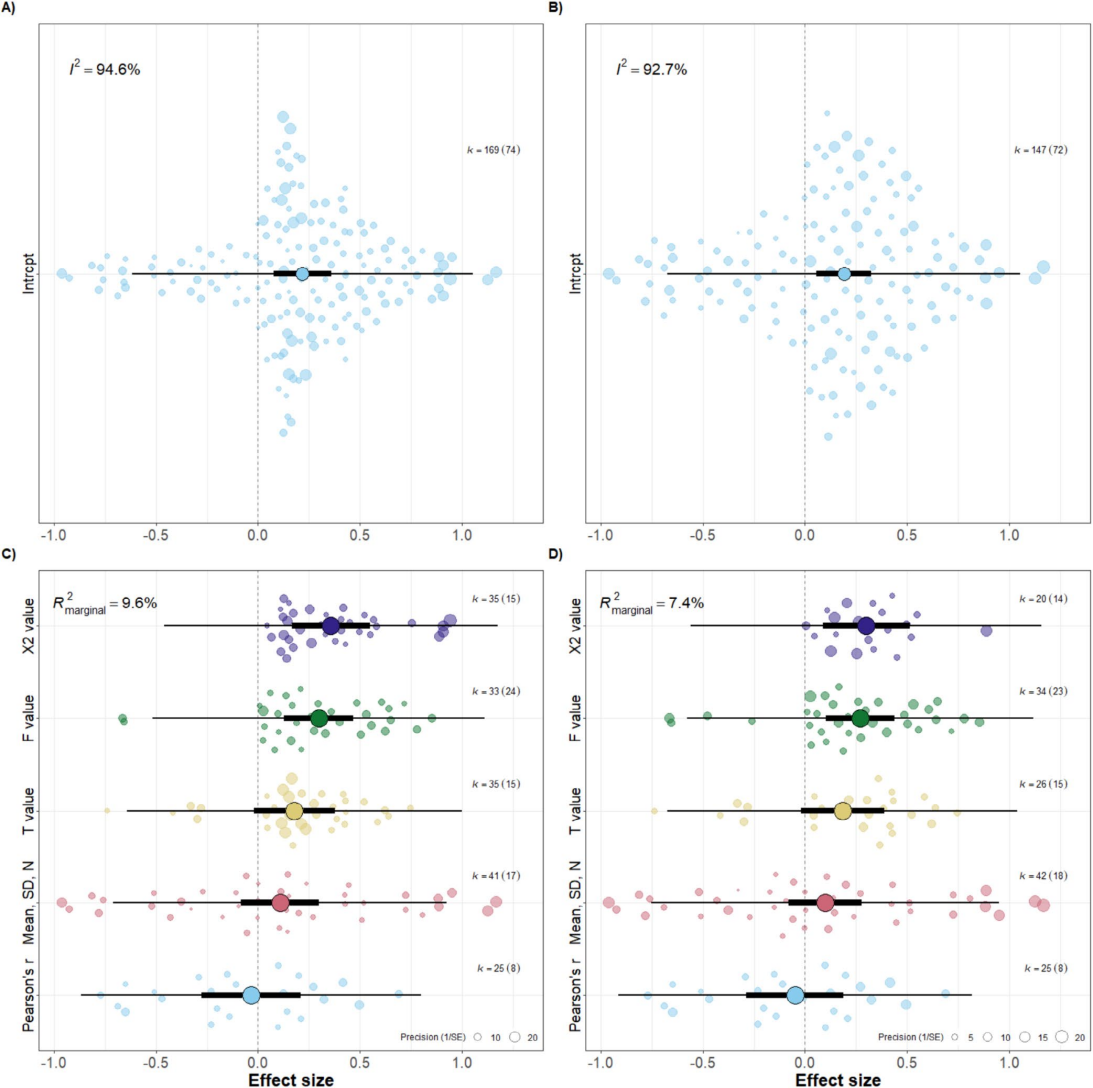
Graphical representation of the relationship between color and aggression after corrections were implemented (A, C) and after data extraction validation of 15% of the studies (B, D). Overall, aggression and coloration are positively associated across species (A, B), but the generality of this relationship is low (i.e., heterogeneity is high), and depends on effect size origin (five levels: *r*, means‐SD‐n [*r*_bis_], *t* values, *F* values, *χ* ^2^ values; C, D), which raises strong methodological concerns. The relationship decreases sensibly after correcting a small fraction (15%) of the data (B, D). Orchard plots of the phylogenetic multilevel meta‐analytic models showing mean overall effects, 95% confidence intervals, 95% prediction intervals and individual effect sizes scaled by their precision (transparent circles). *I^2^* corresponds to relative heterogeneity and *R^2^_marginal_* to the heterogeneity explained by the moderator. *k* is the number of individual effect sizes, the number of studies is shown in brackets.

### 2.6 Statistical Analyses

All phylogenetic multilevel meta‐analyses and meta‐regressions were run using the R package “metafor” v.4.6‐0 (Viechtbauer 2010) in R v.4.3.1 (R Core Team 2023), and included the following random effects: (a) study, which encompasses effect sizes extracted from the same study, (b) species, which encompasses effect sizes derived from the same species, (c) phylogeny, which consists of the phylogenetic correlation matrix between species, and (d) effect size identity, which is a unit‐level effect and represents within‐study variation. We built phylogenetic trees by first searching for and confirming species names in the Open Tree Taxonomy (Rees and Cranston 2017) and then extracting their phylogenetic information from the Open Tree of Life (Hinchliff et al. 2015) using the R package “rotl” v.3.1.0 (Michonneau et al. 2016). We calculated tree branch length following Grafen (1989) with the R package “ape” v.5.7‐1 (Paradis and Schliep 2019) and constructed a phylogenetic correlation matrix for all models. In addition, for our re‐analyses, we specified sampling variances as a variance–covariance matrix rather than a vector of sampling variances to further account for the nonindependence of sampling variances. This variance–covariance matrix had the sampling variance for each effect size on the diagonal and the covariance between these measures, which was calculated assuming a 0.5 correlation between effect size sampling variances from the same study using the function “vcalc()” from the R package “metafor,” as off‐diagonal elements (Noble et al. 2017).

For the intercept‐only models, we performed Cochrane's *Q* test and calculated total absolute heterogeneity (*σ* ^2^) and its source (or relative heterogeneity: *I* ^2^) using the function “i2_ml()” from the R package “orchaRd” v.2.0 (Nakagawa et al. 2023). For the meta‐regressions, we estimated the percentage of heterogeneity explained by the moderator(s) as *R* ^2^_marginal_ (Nakagawa and Schielzeth 2013) using the function “r2_ml()” from the R package “orchaRd.” Throughout, we provide mean estimates with their 95% confidence intervals (CI), 95% prediction intervals (PI), and *p*‐values. While confidence intervals show the likely range for the mean effect, prediction intervals incorporate heterogeneity to estimate the likely range in which 95% of effects are expected to occur for studies similar to those included in the current dataset (IntHout et al. 2016).

## 3 Results

### 3.1 Biased Literature Sampling

We found that none of the 13 published studies included in Sánchez‐Tójar et al. (2018) were identified among the 731 records retrieved at the pre‐screening stage by Ruckman et al. (2024), indicating incomplete search retrieval.

### 3.2 Re‐Calculation of Effect Sizes

The custom function used by Ruckman et al. (2024) to calculate *r*_bis_ led to an artificially narrow range of values (range: *r*_bis.original_ = [−0.64, 1.02]; *r*_bis.fixed_ = [−0.96,1.17]) and an underestimation of the overall correlation (mean and median: *r*_bis.original_ = 0.06 and 0.02; *r*_bis.fixed_ = 0.09 and 0.11; *k* = 42 effect sizes, *r*_bis_ constitute 25% of the dataset). The simulation results further confirmed the large influence that the missing parentheses had on the calculated effect sizes (Figure S1). In addition, these simulations showed that even after adding the missing parentheses, the agreement between the custom function and the function “escalc()” from the R package “metafor” (Viechtbauer 2010) was not perfect (Figure S1), which reinforces our recommendation to use established, reliable functions such as “escalc()” rather than custom functions whenever possible.

### 3.3 Re‐Analysis Non‐Z‐Transformed Effect Sizes (*r* and *r*_bis_): Correct Approach

Our phylogenetic multilevel intercept‐only model using the corrected effect size calculations and their associated sampling variances showed an overall positive and statistically significant correlation (*r* = 0.218, 95% CI = [0.078, 0.359], *p* = 0.003, *k* = 169 effect sizes, *n* = 74 studies, 54 species; Figure 1A). However, contrary to Ruckman et al. (2024), where heterogeneity was reported as virtually non‐existent (*I* ^2^_total_ = 3.3%), our re‐analysis shows high total absolute (*σ* ^2^ = 0.174; *Q* = 4268, *p* < 0.001) and relative heterogeneity (*I* ^2^_total_ = 94.6%). This high heterogeneity leads to wide prediction intervals around the meta‐analytic mean (95% PI = [−0.617, 1.053]), indicating that the statistically significant mean observed has limited generalizability across settings. In addition, the random‐specific *I* ^2^ estimates suggested that most heterogeneity was associated with the within‐study/residual (*I* ^2^_within‐study_ = 49.6%) and phylogeny levels (*I* ^2^_phylogeny_ = 25.7%), followed by differences among studies (*I* ^2^_among‐study_ = 14.8%) and species after accounting for phylogeny (*I* ^2^_species_ = 4.5%).

The phylogenetic multilevel meta‐regression testing for small‐study effects did not show statistically significant evidence of asymmetry in the funnel plot (slope_ESS_ = 0.46, 95% CI = [−0.67, 1.59], *p* = 0.426; *R* ^2^_marginal_ = 0.6%). However, the meta‐analytic mean adjusted for funnel plot asymmetry (i.e., the intercept of the main model) became statistically nonsignificant (*r* = 0.14, 95% CI = [−0.10, 0.37], *p* = 0.247), offering another small note of caution regarding the robustness of the evidence for a positive overall correlation between aggression and coloration.

### 3.4 Effect Sizes Origin

Visually, we found an alarming pattern between effect size origin (i.e., the summary statistics from which they were calculated) and both the percentage of positive effect sizes (*r*: 44%, *r*_bis_: 61%, *t*: 86%, *F*: 94%, *χ* ^2^: 100%) and their magnitude (Figure S2). This suggested that the direction of the effect sizes may not have been appropriately accounted for when data were extracted by Ruckman et al. (2024). In accordance, the phylogenetic multilevel uni‐moderator meta‐regression with effect size origin as the moderator confirmed that results differed largely depending on effect size origin, with results obtained from *χ* ^2^ values leading to the largest average effect (*r* = 0.357, 95% CI = [0.166, 0.549], *p* = < 0.001, *k* = 35 effect sizes, *n* = 15 studies), whereas effects extracted directly from the original studies as Pearson's *r* had an near zero effect (*r* = −0.034, 95% CI = [−0.279, 0.211], *p* = 0.784, *k* = 25 effect sizes, *n* = 8 studies; Figure 1C). The moderator effect size origin explained around one tenth of heterogeneity (*R* ^2^_marginal_ = 9.6%). These differences were reduced after the data extraction validation of 15% of the dataset (see below), but remained substantial (Figure 1D).

### 3.5 Data Extraction Validation

We repeated data extraction for 11 studies (15%) and found issues in 8 studies (73%). We excluded two studies for not meeting the selection criteria (Podberscek and Serpell 1996; Yang et al. 2018), we found additional effect sizes for three studies, some effect sizes were removed from one study for not meeting the selection criteria, the sign of the effect of one study was swapped, the extracted effect size for one study was recalculated, and sample sizes were changed for two studies (note that the same study can have multiple issues). The updated dataset contained 147 effect sizes from 72 studies covering 54 species (*k* = 1–15 effect sizes/species), and we provide a study‐by‐study detailed account of all changes and decisions in “004_data_extraction_validation.qmd” (Sánchez‐Tójar and D'Amelio 2026).

### 3.6 Re‐Analysis After Data Extraction Validation

Solely validating 15% of the extracted primary sources led to a sensibly weaker effect than the original, but still an overall positive and statistically significant correlation in the phylogenetic multilevel intercept‐only model (*r* = 0.191, 95% CI = [0.057, 0.324], *p* = 0.005, *k* = 147 effect sizes, *n* = 72 studies, 54 species; Figure 1B). Total absolute heterogeneity (*σ* ^2^ = 0.186; *Q* = 3652, *p* < 0.001) and relative heterogeneity (*I* ^2^_total_ = 92.7%) were high, and prediction intervals around the meta‐analytic mean remained wide (95% PI = [−0.673, 1.054]), still suggesting that the statistically significant mean has limited generalizability across settings. The random‐specific *I* ^2^ estimates suggested that the most important levels were within‐study/residual (*I* ^2^_within‐study_ = 55.8%) and phylogeny (*I* ^2^_phylogeny_ = 22.2%), followed by differences among studies (*I* ^2^_among‐study_ = 11.7%) and negligible differences among species after accounting for phylogeny (*I* ^2^_species_ = 3.0%).

The phylogenetic multilevel meta‐regression testing for small‐study effects showed no statistical evidence of asymmetry in the funnel plot (slope_SEZr_ = −0.11, 95% CI = [−1.46, 1.24], *p* = 0.874; *R* ^2^_marginal_ = 0.02%). However, as before, the meta‐analytic mean adjusted for funnel plot asymmetry, which was statistically nonsignificant (*r* = 0.21, 95% CI = [−0.06, 0.48], *p* = 0.127), confirmed the additional note of caution regarding the robustness of the evidence for a positive overall correlation between aggression and coloration.

## 4 Discussion

Rohwer's badge of status hypothesis was developed to explain why the plumage of some bird species is highly variable while in others it is not (Rohwer 1975). Fifty years after its introduction, the validity and generality of this hypothesis across species remain unresolved, as already suggested by a re‐evaluation of what was long considered its textbook example, the house sparrow ( *Passer domesticus* ; Sánchez‐Tójar et al. 2018). In this letter, we show that methodological concerns and errors in Ruckman et al. (2024) preclude meaningful interpretation of their findings. Rather than proposing an alternative explanation, we argue that a new, more rigorous data collection effort is needed to assess the general relationship between aggression and coloration. Below, we outline several key issues and offer recommendations to help prevent similar errors in future work.

### 4.1 Data Collection Targeted to the Hypothesis

In evidence synthesis, ensuring comprehensive and appropriate literature sampling for the hypothesis under investigation is essential for a valid interpretation of the findings. We fully acknowledge that systematic reviews and meta‐analyses are particularly difficult in ecology and evolution (Stewart and Schmid 2015): searching for ecological and evolutionary literature is challenging, particularly because the scope of the questions is usually broad, encompassing multiple species, methods, habitats, and outcomes. This is further exacerbated by a lack of standardization in terminology in many subfields.

Our assessment suggests that the search in Ruckman et al. (2024) was incomplete and inappropriate to test the badge of status hypothesis. Sampling should be rooted in the biological premises of the hypothesis tested. For example, Rohwer's original badge of status hypothesis was embedded in the sociality of the species (Rohwer 1975), but this aspect is neglected in the current synthesis, which includes animals without a visual system (e.g., anemones) as well as nocturnal, color‐blind animals that likely do not rely on visual communication during agonistic interactions (i.e., *Mus musculus* ). Moreover, we argue that testing the evolutionary significance of the badge of status hypothesis should not include domestic animals (e.g., dogs), as artificial selection creates color morphs not present in the wild, as well as altering behavior and morphology in general. In addition, although we acknowledge the difficulty of it, literature searches should strive for completeness, which was not the case in Ruckman et al. (2024). For example, although Sánchez‐Tójar et al. (2018)'s meta‐analysis on the badge of status hypothesis in house sparrows is cited several times in Ruckman et al. (2024), the two meta‐analyses included entirely different primary studies (i.e., zero overlap). The limited keyword set used in Ruckman et al. (2024)'s search string, omitting central terms such as “dominance,” “status,” or “badge,” likely produced a literature sample that does not represent the body of evidence available on the hypotheses that Ruckman et al. (2024) set to test. To minimize these biases, we recommend comparing search results and study selection with those of previous meta‐analyses, thereby ensuring continuity and cumulative evidence building. In addition, using “reference studies” (e.g., key, foundational papers that should be retrieved by any comprehensive search) helps verify that the literature sampling is sufficiently complete and aligned with existing knowledge in the field before proceeding with the data extraction and synthesis. For a practical guide to systematic searching and study screening for literature reviews in ecology and evolution, we recommend the detailed guides by Foo et al. (2021) and Lagisz et al. (2025).

### 4.2 Extraction, Calculation of Effect Sizes and Heterogeneity

We found a mistake in the custom function used to calculate biserial correlations (*r*_bis_). We suggest using the existing and reliable function “escalc()” from the R package “metafor” (Viechtbauer 2010) rather than writing the function oneself. In addition, we caution against performing a Fisher Z‐transformation when a meta‐analysis combines both Pearson's correlation coefficients (*r*) and *r*_bis_, as recommended by the authors of the methodological study on this topic (Jacobs and Viechtbauer 2017). While correcting these mistakes did not lead to a strong difference in the overall results, more impactful was the miscalculation of the heterogeneity, from an *I* ^2^ of 3.3% in Ruckman et al. (2024) to our estimate of 94.6%. Heterogeneity corresponds to the variance in the “true” effects and is a measurement that allows us to understand inconsistency among effect sizes (or, said differently, the generalizability of the meta‐analytic mean). Heterogeneity is often high in ecology and evolution (Senior et al. 2016; Yang, Noble, Spake, et al. 2025) and meta‐analyses should always account for it by calculating prediction intervals and, importantly, taking them into account in the results interpretation (Spake et al. 2022, 2023; Usui et al. 2021; Yang, Noble, Senior, et al. 2025). Finding low heterogeneity should be surprising in this field and should be treated as a possible red flag. For a theoretical and practical guide to calculating and interpreting heterogeneity in ecology and evolution, we recommend following the pluralistic approach suggested by Yang, Noble, Spake, et al. (2025). In addition, after observing that the evidence differed depending on the statistical origin of the effect sizes, we performed a validation check on 15% of the original dataset. This verification revealed disagreements in data extraction in 73% of cases, highlighting errors and inconsistencies in the original extraction. We highlight three points summarizing several of the issues studied that future meta‐analytic authors should pay particular attention to: (a) inherently unsigned test statistics (*F*, *χ* ^2^) should be treated with care as they can be mistakenly assigned as positive by default, (b) traits indicating the same relationship can have opposite signs (e.g., in latency to approach higher values indicate lower aggression), and (c) possible selective reporting of statistically significant results can lead to a sign bias. A striking example of this case is a study on domestic dogs originally included (Podberscek and Serpell 1996), in which the original authors presented only the single statistically significant effect size on aggression, thereby skewing the data. These results underscore the importance of double extraction or independent verification as a routine quality‐control step, an approach that was not reported to have been used in Ruckman et al. (2024).

While even after our corrections the overall posterior effect size showed a statistically positive correlation, re‐extracting just 15% of the primary sources caused a decrease of 23% of the mean effect size (from 0.248 to 0.191). Moreover, accounting for the high heterogeneity substantially changes the interpretation (Figure 1A,B), indicating that the statistically significant mean effect has limited generalizability across settings. Although small‐study effects tests did not provide clear evidence for funnel plot assymetry, they added further nuance. Moreover, we observed an alarming pattern linking effect size origin (i.e., the summary statistics from which effect sizes were derived) to both the proportion of positive effects (*r*: 44%, *r*_bis_: 61%, *t*: 86%, *F*: 94%, *χ* ^2^: 100%) and their magnitude, which our analyses confirmed. Estimates derived directly from correlations and mean comparisons were statistically nonsignificant and differed substantially from those obtained by transforming inferential statistics (*t*, *F*, *χ* ^2^). This discrepancy raises strong methodological concerns about the comparability and reliability of effect sizes across sources.

### 4.3 Conclusions

How aggression and coloration are related is unlikely to have widely homologous patterns across the phylogenetic tree, even within coloration types. Given the diversity of synthesis pathways, pigment origin, and pleiotropy of the genetic machinery involved, it is unlikely that a unique mechanism is shared among all species (Britton and Davidowitz 2023; Hill et al. 2023). This might be at the foundation of the high level of heterogeneity found in our re‐analysis, including the relatively high importance of phylogeny. Alongside efforts to identify the physiological pathways of color synthesis, we argue that more species‐specific research is needed to quantify the extent of color variation, determine how it is perceived by conspecifics, and once these conditions are met, assess whether signaling is honest (i.e., condition‐dependent) and whether associated costs and trade‐offs exist. We argue that improving taxonomic representation in high‐quality studies is necessary to uncover general patterns. Future primary research and meta‐analyses should strive to incorporate this complexity.

Although our letter is critical, we would not like to finish it without commending the original authors for their openness and for sharing their data and code. Data‐ and code‐sharing are becoming increasingly common in ecology and evolution and are rapidly becoming the norm (Ivimey‐Cook et al. 2025). Our study illustrates how such openness can facilitate post‐publication error correction (Berberi and Roche 2022; Chen et al. 2023) and help to normalize post‐publication peer review (Kriegeskorte 2012). In this context, routine code review, implemented early in projects (Ivimey‐Cook et al. 2023) and embedded within peer review through roles such as data editors (Pick et al. 2026), should be seen as a natural extension of emerging open science norms.

## Author Contributions

**Alfredo Sánchez‐Tójar:** conceptualization (equal), data curation (equal), formal analysis, investigation (equal), methodology (equal), project administration (equal), software (equal), visualization (equal), writing – original draft (equal), writing – review and editing (equal). **Pietro B. D'Amelio:** conceptualization (equal), data curation (equal), investigation (equal), methodology (equal), project administration (equal), software (equal), validation (equal), writing – original draft (equal), writing – review and editing (equal).

## Funding

A.S.‐T. was partially supported by Portuguese national funds via Fundação para a Ciência e a Tecnologia (FCT) under projects LA/P/0058/2020, UID/PRR/4539/2025, and UID/04539/2025, and project EXCELScIOR, funded by the EU's Horizon Europe under Grant Agreement No. 101087416. The funders had no role in the study design, data collection and analysis, decision to publish, or the preparation of the manuscript.

## Conflicts of Interest

Both authors have been actively engaged in research on this topic and have an ongoing pre‐registered meta‐analysis project (pre‐registration date: July 22, 2024; https://doi.org/10.17605/OSF.IO/JW6YZ), which may be considered a potential conflicts of interest.

## Supporting information


**Data S1:** ece373578‐sup‐0001‐Figures.docx.

**Data S2:** ece373578‐sup‐0002‐Supinfo.html.

## Data Availability

All data and code are available in Zenodo at https://doi.org/10.5281/zenodo.19628290 (Sánchez‐Tójar and D'Amelio 2026) and at https://github.com/ASanchez‐Tojar/meta‐analysis_badge_of_status_commentary. The data re‐analyzed here were originally compiled by Ruckman et al. (2024) and shared publicly at Dryad (https://doi.org/10.5061/dryad.9kd51c5tk).
