# Supplementary material for: Fifty Years Later, and We Still Don't Know About Badges of Status
Source: Ecol Evol. 2026 Apr 29;16(5):e73578. doi: 10.1002/ece3.73578 (PMC13129410; doi:10.1002/ece3.73578)
Supplement: Supplementary file 1 — Data S1: ece373578‐sup‐0001‐Figures.docx. [file ECE3-16-e73578-s002.docx]

**Supplementary figures**


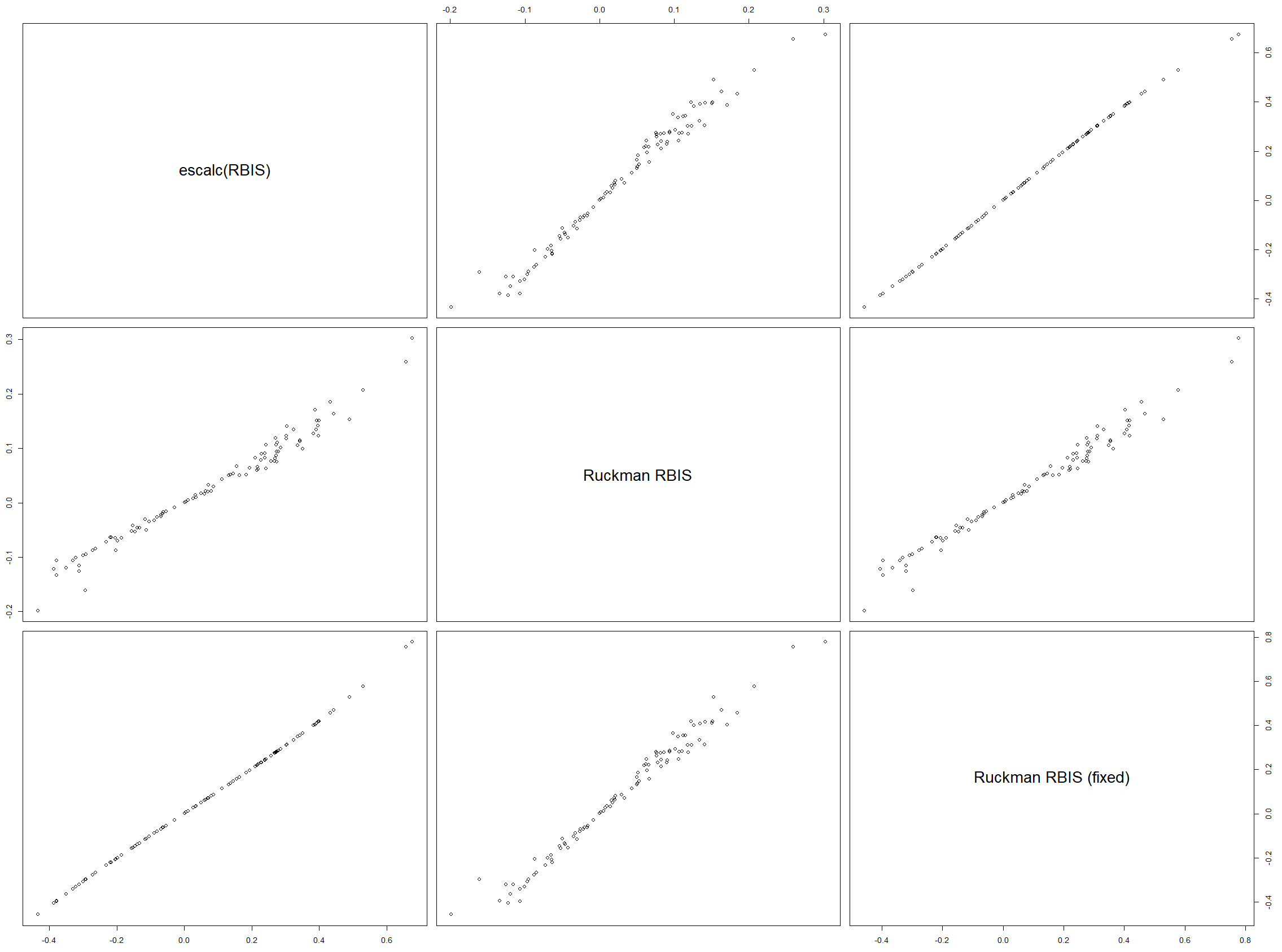


**Figure S1**. Pairwise correlation plot showing the agreement between three methods for calculating biserial correlations (*r_bis_*) using simulations: the approach originally used by Ruckman et al., (2024; "Ruckman RBIS"), a corrected version of their approach ("Ruckman RBIS (fixed)"), and the method used in our re-analysis via the ‘escalc()’ function from the R package ‘metafor’.


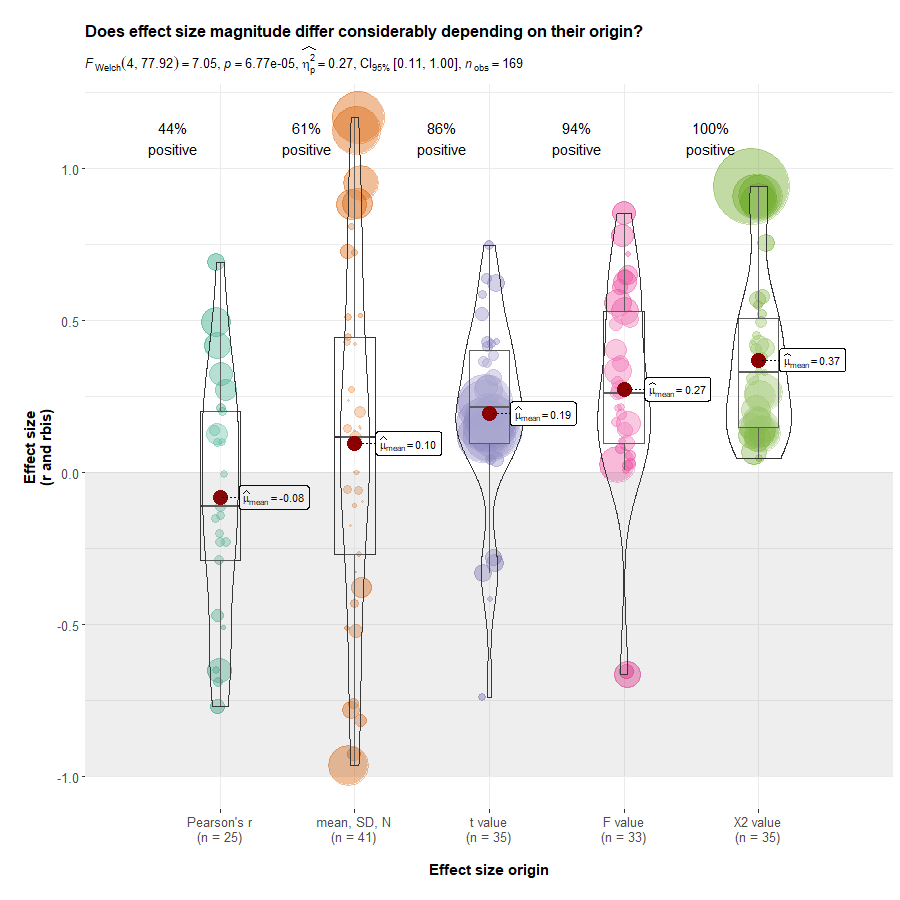

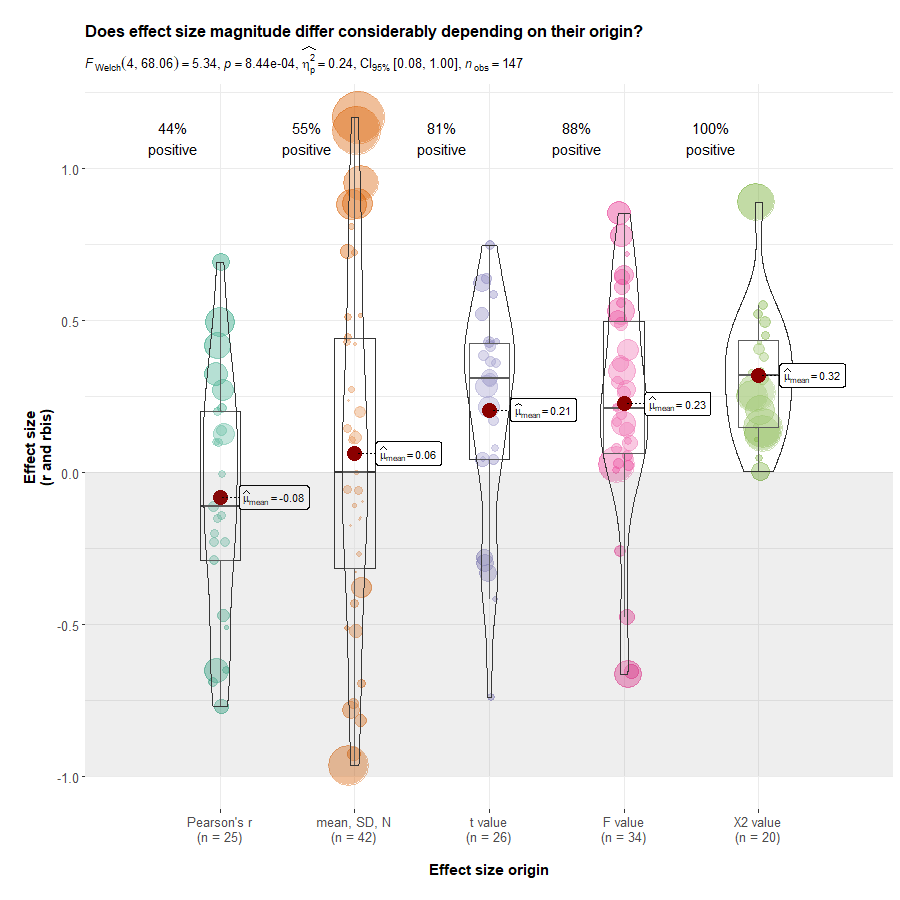


**Figure S2**. Effect size magnitude differs depending on the origin of the effect size, with effect sizes obtained directly as Pearson’s *r* being, on average, the smallest and effect sizes obtained by transforming *χ^2^* values being, on average, the largest. In addition, the percentage of positive (or zero) effect sizes differ strongly between different origins, with effect sizes obtained from *χ^2^* values being positive at all times, which suggests that effect size direction may not have been properly adjusted. Left-hand and right-hand side panel corresponds to the results before and after data extraction validation, respectively. The figure shows individual effect sizes scaled by their precision (i.e., 1/SE; coloured data points, w), mean (red circle), boxplot shows median and corresponding 25th and 75th quartiles (squared box), violin outline shows the distribution of the raw data, and central line shows min and max values. The *F* test shown corresponds to Welch’s one-way ANOVA, *η^2^_p_* is the corresponding partial eta squared, *µ* corresponds to the raw arithmetic mean, and *n* in this figure refers to the number of effect sizes. Figure generated using the function ‘ggbetweenstats’ from the R package ‘ggstatsplot’ v. 0.12.5
