# Supplementary material for: Fifty Years Later, and We Still Don't Know About Badges of Status
Source: Ecol Evol. 2026 Apr 29;16(5):e73578. doi: 10.1002/ece3.73578 (PMC13129410; doi:10.1002/ece3.73578)
Supplement: Supplementary file 2 — Data S2: ece373578‐sup‐0002‐Supinfo.html. [file ECE3-16-e73578-s001.html]

Supporting Information for: Fifty years later, and we still don’t know about badges of status


## Table of contents

- Importing original new dataset
  - Andrews et al. 1996
  - Carola et al. 2014
  - Naretto and Chiaraviglio 2023
  - Martin and Hengstebeck 1981
  - Dijkstra et al 2009b
  - Yang et al 2018
  - Rose and Soole 2020
  - Podberscek and Serpell 1996
  - Karlsson et al. 2011
  - Seaver and Hurd 2017
  - Zinzow-Kramer et al. 2015
- Describing differences between original and updated dataset
- Conclusions
  - R session info

# Supporting Information for: Fifty years later, and we still don’t know about badges of status

 Code

- Show All Code
- Hide All Code
- ---
- View Source

Author

Alfredo Sanchez-Tojar, Pietro B. D’Amelio

# Importing original new dataset

This corresponds to the dataset containing all recalculated effect sizes generated by script ‘001\_effect\_size\_calculation.R’.

Code

```
metadata.new <- read.csv("../data/new/meta_complete_data2_new.csv", header=T)

#excluding it here already because the authors decided to exclude from the final analyses
metadata.new <- metadata.new %>%
  filter(Classification!="pteridine")

# creating a copy for fixing and adding effect sizes
metadata.new.updated <- metadata.new

# list of columns of interest for re-extracting and adding new effect sizes
columns.of.interest <- c("Authors","Publication.Year","Study","Species",
                         "Sample.Size","Stat.Test","Test.Statistic","df1","df2",
                         "r","n1","mean1","sd1","n2","mean2","sd2","yi","vi")


knitr::kable(head(metadata.new[,-c(1:2)]),format = "html")
```

| Authors | Publication.Year | Study | Species | Geographic | Vert\_Invert | Color1 | Color2 | Color3 | Pattern | Classification | Eu\_Pheomelanin | Social\_Rank\_Controlled | Obs\_vs\_Exp | Condition\_Stats | Condition | Age | Sex | Location | Season | Plasticity | Aggression | Aggression.Units | Sample.Size | Stat.Test | Test.Statistic | df1 | df2 | p.value | r | n1 | mean1 | var1 | se1 | sd1 | n2 | mean2 | var2 | se2 | sd2 | yi | vi |
| --- | --- | --- | --- | --- | --- | --- | --- | --- | --- | --- | --- | --- | --- | --- | --- | --- | --- | --- | --- | --- | --- | --- | --- | --- | --- | --- | --- | --- | --- | --- | --- | --- | --- | --- | --- | --- | --- | --- | --- | --- | --- |
| Lehtonen, TK | 2014 | Lehtonen 2014 - 1 | Amphilophus sagittae | Crater Lake Xiloa, Nicaragua | vertebrate | gold |  | dark | body | melanocortin | eumelanin | dummy used | Exp | Covariate | Length | mature | males | field | breeding | No | Direct | rate/5 min | 45 | mean | NA | NA | NA | NA | NA | 21 | 2.655 | NA | NA | 2.8620000 | 24 | 3.070 | NA | NA | 2.207000 | 0.1045729 | 0.0351715 |
| Lehtonen, TK | 2014 | Lehtonen 2014 - 2 | Amphilophus sagittae | Crater Lake Xiloa, Nicaragua | vertebrate | gold |  | dark | body | melanocortin | eumelanin | dummy used | Exp | Covariate | Length | mature | females | field | breeding | No | Direct | rate/5 min | 38 | mean | NA | NA | NA | NA | NA | 15 | 3.463 | NA | NA | 2.6770000 | 23 | 4.446 | NA | NA | 1.928000 | 0.2721993 | 0.0386500 |
| Clement, TS; Parikh, V; Schrumpf, M; Fernald, RD | 2005 | Clement et al 2005 | Astatotilapia burtoni | Lake Tanaganyika, Tanzania | vertebrate | drab blue/yellow |  | bright blue/yellow | body | carotenoid | N/A | video | Exp | NS (F1,10=0.628, p = 0.451) | Size Matched -activity level same | mature | males | lab | year round | Plastic | Direct | territorial | 28 | mean | NA | NA | NA | NA | NA | 5 | 0.382 | NA | 0.144 | 0.3220000 | 5 | 0.732 | NA | 0.028 | 0.063000 | 0.8079880 | 0.0405434 |
| Renn, SCP; Fraser, EJ; Aubin-Horth, N; Trainor, BC; Hofmann, HA | 2012 | Renn et al 2012 | Astatotilapia burtoni | Lake Tanaganyika, Tanzania | vertebrate | no black |  | black | face | melanocortin | eumelanin | uncontrolled | Obs | Size matched | Weight and length | mature | females | lab | year round | No | Direct | number of chases | 36 | mean | NA | NA | NA | NA | NA | 21 | 0.840 | NA | 0.160 | 0.7332121 | 15 | 11.460 | NA | 0.860 | 3.330766 | 1.1689200 | 0.0022032 |
| Boerner, M; Kruger, O | 2009 | Boerner and Kruger 2009 - 1 | Buteo buteo | Westphalia, Germany | vertebrate | light | intermediate | dark | body | melanocortin | eumelanin | dummy used | Exp | uncontrolled | None measured | mature | males | field | breeding | No | Indirect | score | 54 | mean | NA | NA | NA | NA | NA | 16 | 1.728 | NA | 0.141 | 0.5640000 | 7 | 0.570 | NA | 0.368 | 0.973600 | -0.8151808 | 0.0195386 |
| Boerner, M; Kruger, O | 2009 | Boerner and Kruger 2009 - 2 | Buteo buteo | Westphalia, Germany | vertebrate | light | intermediate | dark | body | melanocortin | eumelanin | dummy used | Exp | uncontrolled | None measured | mature | females | field | breeding | No | Indirect | score | 54 | mean | NA | NA | NA | NA | NA | 20 | 0.632 | NA | 0.176 | 0.7871000 | 4 | 1.710 | NA | 0.255 | 0.510000 | 0.7243681 | 0.0402852 |

In the following sections, we reassess the data extraction of 11 studies, which correspond to 15% of all studies included in the meta-analysis of Ruckman et al. (2024).

## Andrews et al. 1996

This study appears in the dataset as:

Code

```
Andrews_et_al_1996_subset <- metadata.new %>% filter(Authors=="Andrews, TJ; Summers, CH") %>% select(all_of(columns.of.interest))

knitr::kable(Andrews_et_al_1996_subset,format = "html")
```

| Authors | Publication.Year | Study | Species | Sample.Size | Stat.Test | Test.Statistic | df1 | df2 | r | n1 | mean1 | sd1 | n2 | mean2 | sd2 | yi | vi |
| --- | --- | --- | --- | --- | --- | --- | --- | --- | --- | --- | --- | --- | --- | --- | --- | --- | --- |
| Andrews, TJ; Summers, CH | 1996 | Andrews and Summers 1996 - 1 | Anolis carolinensis | 18 | t | 1.920 | 16 | NA | NA | NA | NA | NA | NA | NA | NA | 0.4327311 | 0.0388560 |
| Andrews, TJ; Summers, CH | 1996 | Andrews and Summers 1996 - 2 | Anolis carolinensis | 18 | F | 16.393 | 5 | 53 | NA | NA | NA | NA | NA | NA | NA | 0.7792981 | 0.0090711 |

For this species (i.e., Anolis carolinensis), Ruckman et al. (2024) classified *Light green* as the “Light Color” and *Dark green or Brown* as the “Dark Color”. Two effect sizes were extracted.

The *t* value was extracted from following original text: “*Dominant and subordinate females were also not significantly (t16 = 1.92, p > 0.072) different in mean body color in the absence of a male; all visible pigmented body surface of both females were a light to moderate green*”

- **Our assessment**: The *t* value comes from an independent *t* test comparing subordinate vs dominant for the no male condition. Performed data extraction is clear.
- **Required action**: None.

The F value was extracted from following original text: “*However, when males were present body coloration was significantly darker in all females, and statistically darkest in dominant or single females (F5,53 = 16.393, p < 0.001)*”

- **Our assessment**: The *F* value seems to correspond to the following ANOVA: “*Comparisons were made statistically for aggressive, submissive and courtship behavior, perch site selection and color by paired t-test or ANOVA*”), which contains 2 predictors: (1) context (levels: no male, male present), and treatment (levels: single, subordinate, dominant), which explain why df1 = 5. The reason for df2 = 53 is because there are 9 females in each group for a total of 54. Since 18 rather than 54 is used as the sample size when calculating *Vr*, there is no action required for this study.
- **Required action**: None.

## Carola et al. 2014

This study appears in the dataset as:

Code

```
Carola_et_al_2014_subset <- metadata.new %>% filter(Authors=="Carola, V; Perlas, E; Zonfrillo, F; Soini, HA; Novotny, MV; Gross, CT") %>% select(all_of(columns.of.interest))

knitr::kable(Carola_et_al_2014_subset,format = "html")
```

| Authors | Publication.Year | Study | Species | Sample.Size | Stat.Test | Test.Statistic | df1 | df2 | r | n1 | mean1 | sd1 | n2 | mean2 | sd2 | yi | vi |
| --- | --- | --- | --- | --- | --- | --- | --- | --- | --- | --- | --- | --- | --- | --- | --- | --- | --- |
| Carola, V; Perlas, E; Zonfrillo, F; Soini, HA; Novotny, MV; Gross, CT | 2014 | Carola et al 2014 | Mus musculus | 20 | F | -5.4 | 1 | 18 | NA | NA | NA | NA | NA | NA | NA | -0.6546537 | 0.0171858 |

For this species (i.e., Mus musculus), Ruckman et al. (2024) classified *Non-agouti* as the “Light Color” and *Agouti* as the “Dark Color”. Note that Ruckman et al. (2024) wrote that aggression between different morphs were said to be excluded “we therefore limit our data set to measure of aggression within color morphs”. One effect size was extracted.

The F value was extracted from following original text: “*Non-agouti mice showed significantly increased aggressive-like behavior when compared to agouti littermates in the test, exhibiting more attacks [Figure 1A; repeated measure ANOVA, genotype effect: F1,18 = 5.40, P = 0.032]*”

- **Our assessment**: Since the Non-agouti is considered the “Light Color”, the sign of the final effect size should be negative, which is.
- **Required action**: None.

When revisiting this study, we realized that there was an additional aggression proxy that was not extracted: “*…and a shorter latency to the first attack [Figure 1B; repeated measure ANOVA, genotype effect: F1,18 = 7.77; P = 0.012] toward a non-agouti intruder over three consecutive trials.*”.

- **Our assessment**: There is no clear reason why this proxy was not extracted since latency was extracted for other studies in the dataset. In the full dataset there are several other papers where multiple effect sizes from the same group of animals were extracted. This sort of nonindependence (i.e., multiple estimates from the same group of animals) should be accounted for with a random effect (i.e., “Group ID”). This should be done for all such cases in the dataset.
- **Required action**: We extracted the data for the additional effect size from Figure 1B using the R package `metaDigitise` (Pick et al. 2019). The corresponding *rbis* are calculated below and then added to the database.

Code

```
Carola_et_al_2014_extra_1 <- metadata.new %>% filter(Authors=="Carola, V; Perlas, E; Zonfrillo, F; Soini, HA; Novotny, MV; Gross, CT")

# emptying entry
Carola_et_al_2014_extra_1[,c(1:ncol(Carola_et_al_2014_extra_1))] <- NA

# adding variables of interest from original sources
Carola_et_al_2014_extra_1[,c("Authors","Publication.Year","Species")] <- Carola_et_al_2014_subset[,c("Authors","Publication.Year","Species")]

Carola_et_al_2014_extra_1[,"Study"] <- "Carola et al 2014 - 2"
Carola_et_al_2014_extra_1[,"Sample.Size"] <- 20
Carola_et_al_2014_extra_1[,"Stat.Test"] <- "mean"
Carola_et_al_2014_extra_1[,"Test.Statistic"] <- 7.77
Carola_et_al_2014_extra_1[,"df1"] <- 1
Carola_et_al_2014_extra_1[,"df2"] <- 18
Carola_et_al_2014_extra_1[,"n1"] <- 10
Carola_et_al_2014_extra_1[,"mean1"] <- 22.94877
Carola_et_al_2014_extra_1[,"sd1"] <- 10.578670
Carola_et_al_2014_extra_1[,"n2"] <- 10
Carola_et_al_2014_extra_1[,"mean2"] <- 35.74442
Carola_et_al_2014_extra_1[,"sd2"] <- 9.785269

# caculating rbis
Carola_et_al_2014_extra_1 <- as.data.frame(escalc(measure = "RBIS",
                                                  n2i = n1,
                                                  n1i = n2,
                                                  m2i = mean1,
                                                  m1i = mean2,
                                                  sd2i = sd1,
                                                  sd1i = sd2,
                                                  data = Carola_et_al_2014_extra_1))

# flipping the sign to reflect that it is the Non-agouti (Light Color) that takes less time to attack
Carola_et_al_2014_extra_1[,"yi"] <- Carola_et_al_2014_extra_1[,"yi"] * (-1)

# finally, adding this entry to the new dataset
metadata.new.updated <- rbind(metadata.new.updated,Carola_et_al_2014_extra_1)
```

There seem to be several other effect sizes that could have been extracted from this paper: “*To evaluate if aggressive behavior of the resident could be modulated by the genotype of the intruder a fourth trial was carried out in which each group was split and half were exposed to non-agouti and the other half to agouti intruders. No significant behavioral differences between mice exposed to agouti or non-agouti intruders were detected (Figure S2)*”.

## Naretto and Chiaraviglio 2023

This study appears in the dataset as:

Code

```
Naretto_and_Chiaraviglio_2023_subset <- metadata.new %>% filter(Authors=="Naretto, Sergio; Chiaraviglio, Margarita") %>% select(all_of(columns.of.interest))

knitr::kable(Naretto_and_Chiaraviglio_2023_subset,format = "html")
```

| Authors | Publication.Year | Study | Species | Sample.Size | Stat.Test | Test.Statistic | df1 | df2 | r | n1 | mean1 | sd1 | n2 | mean2 | sd2 | yi | vi |
| --- | --- | --- | --- | --- | --- | --- | --- | --- | --- | --- | --- | --- | --- | --- | --- | --- | --- |
| Naretto, Sergio; Chiaraviglio, Margarita | 2023 | Naretto et al 2023 | Pristidactylus achalensis | 46 | F | -6.88 | 2 | 45 | NA | NA | NA | NA | NA | NA | NA | -0.6636723 | 0.0069574 |

For this species (i.e., Pristidactylus achalensis), Ruckman et al. (2024) classified *Lighter* as the “Light Color” and *Darker* as the “Dark Color”. One effect size was extracted.

The extracted *F* value corresponds to the following: “*Average lightness was higher in winners compared to both losers and males categorized as having no clear outcome in the first two rounds of the tournament (Table 2; Round 1 F2,45 = 6.88, P = 0.002)*”

- **Our assessment**: Since the Lighter are the “Light Color”, the sign of the final effect size should indeed be negative, which is. Nonetheless, the sample size should be 48 (i.e., 17+14+17) instead of 46 according to Table 2.
- **Required action**: Change the sample size to 48, and recalculate *vi* to account for this change.

Code

```
metadata.new.updated[metadata.new.updated$Study=="Naretto et al 2023","Sample.Size"] <- 48

yi.tmp <- metadata.new.updated[metadata.new.updated$Study=="Naretto et al 2023","yi"]
Sample.Size.tmp <- metadata.new.updated[metadata.new.updated$Study=="Naretto et al 2023","Sample.Size"]

metadata.new.updated[metadata.new.updated$Study=="Naretto et al 2023","vi"] <- ((1-(yi.tmp^2))^2)/(Sample.Size.tmp-1)
```

When revisiting this study, we realized that there were additional effect sizes that could have been extracted corresponding to Rounds 2 and 3: “*…; Round 2 F2,39 = 5.752, P = 0.006; Round 3 F2,37 = 1.344, P = 0.273)*”.

- **Our assessment**: Those two effect sizes come from the same group of animals. In the full dataset there are several other papers where multiple effect sizes from the same group of animals were extracted. This sort of nonindependence (i.e., multiple estimates from the same group of animals) should be accounted for with a random effect (i.e., “Group ID”). This should be done for all such cases in the dataset.
- **Required action**: To extract these effect sizes, we first confirm that indeed the direction should remain negative by checking Table 2, and then transforming those two *F* values as we did for the *F* value above.

Code

```
Naretto_and_Chiaraviglio_2023_extra_1 <- metadata.new %>% filter(Authors=="Naretto, Sergio; Chiaraviglio, Margarita")

Naretto_and_Chiaraviglio_2023_extra_2 <- metadata.new %>% filter(Authors=="Naretto, Sergio; Chiaraviglio, Margarita")

# emptying entry
Naretto_and_Chiaraviglio_2023_extra_1[,c(1:ncol(Naretto_and_Chiaraviglio_2023_extra_1))] <- NA

Naretto_and_Chiaraviglio_2023_extra_2[,c(1:ncol(Naretto_and_Chiaraviglio_2023_extra_2))] <- NA


# adding variables of interest from original sources
Naretto_and_Chiaraviglio_2023_extra_1[,c("Authors","Publication.Year","Species")] <- Naretto_and_Chiaraviglio_2023_subset[,c("Authors","Publication.Year","Species")]

Naretto_and_Chiaraviglio_2023_extra_2[,c("Authors","Publication.Year","Study","Species")] <- Naretto_and_Chiaraviglio_2023_subset[,c("Authors","Publication.Year","Study","Species")]

# Round 2 value
Naretto_and_Chiaraviglio_2023_extra_1[,"Study"] <- "Naretto et al 2023 - 2"
Naretto_and_Chiaraviglio_2023_extra_1[,"Sample.Size"] <- (9+24+9)
Naretto_and_Chiaraviglio_2023_extra_1[,"Stat.Test"] <- "F"
Naretto_and_Chiaraviglio_2023_extra_1[,"Test.Statistic"] <- 5.752
Naretto_and_Chiaraviglio_2023_extra_1[,"df1"] <- 2
Naretto_and_Chiaraviglio_2023_extra_1[,"df2"] <- 39

# caculating corresponding r
df1.tmp <- Naretto_and_Chiaraviglio_2023_extra_1[,"df1"]
df2.tmp <- Naretto_and_Chiaraviglio_2023_extra_1[,"df2"]
Test.Statistic.tmp <- Naretto_and_Chiaraviglio_2023_extra_1[,"Test.Statistic"]

Naretto_and_Chiaraviglio_2023_extra_1[,"yi"] <- sqrt((df1.tmp*Test.Statistic.tmp)/(df1.tmp*Test.Statistic.tmp+df2.tmp))

# adjusting the sign accordingly 
Naretto_and_Chiaraviglio_2023_extra_1[,"yi"] <- Naretto_and_Chiaraviglio_2023_extra_1[,"yi"]*(-1)

# calculating vi
yi.tmp <- Naretto_and_Chiaraviglio_2023_extra_1[,"yi"]
Sample.Size.tmp <- Naretto_and_Chiaraviglio_2023_extra_1[,"Sample.Size"]
Naretto_and_Chiaraviglio_2023_extra_1[,"vi"] <- ((1-(yi.tmp^2))^2)/(Sample.Size.tmp-1)

# Round 3 value
Naretto_and_Chiaraviglio_2023_extra_2[,"Study"] <- "Naretto et al 2023 - 3"
Naretto_and_Chiaraviglio_2023_extra_2[,"Sample.Size"] <- (5+30+5)
Naretto_and_Chiaraviglio_2023_extra_2[,"Stat.Test"] <- "F"
Naretto_and_Chiaraviglio_2023_extra_2[,"Test.Statistic"] <- 1.344
Naretto_and_Chiaraviglio_2023_extra_2[,"df1"] <- 2
Naretto_and_Chiaraviglio_2023_extra_2[,"df2"] <- 37

# calculating corresponding r
df1.tmp <- Naretto_and_Chiaraviglio_2023_extra_2[,"df1"]
df2.tmp <- Naretto_and_Chiaraviglio_2023_extra_2[,"df2"]
Test.Statistic.tmp <- Naretto_and_Chiaraviglio_2023_extra_2[,"Test.Statistic"]

Naretto_and_Chiaraviglio_2023_extra_2[,"yi"] <- sqrt((df1.tmp*Test.Statistic.tmp)/(df1.tmp*Test.Statistic.tmp+df2.tmp))

# adjusting the sign accordingly 
Naretto_and_Chiaraviglio_2023_extra_2[,"yi"] <- Naretto_and_Chiaraviglio_2023_extra_2[,"yi"]*(-1)

# calculating vi
yi.tmp <- Naretto_and_Chiaraviglio_2023_extra_2[,"yi"]
Sample.Size.tmp <- Naretto_and_Chiaraviglio_2023_extra_2[,"Sample.Size"]
Naretto_and_Chiaraviglio_2023_extra_2[,"vi"] <- ((1-(yi.tmp^2))^2)/(Sample.Size.tmp-1)

# finally, adding this entry to the new dataset
metadata.new.updated <- rbind(metadata.new.updated,
                              Naretto_and_Chiaraviglio_2023_extra_1,
                              Naretto_and_Chiaraviglio_2023_extra_2)
```

In addition, the study also provides three additional tests corresponding to differences in lightness before the trials: “*There were no significant differences in lightness before the beginning of each trial between categories (Table 2; Opponent A and Opponent B in Round 1: F 1,46 = 0.003, P = 0.955; W, NCO and L in Round 2: F2,39 = 1.604, P = 0.214; W, NCO and L in Round 3: F 2,37 = 0.661, P = 0.523).*”.

- **Our assessment**: From what is provided, we consider these set of three effect sizes alternative to the three already extracted, meaning that there is a reasonable argument for deciding whether to extract the effect sizes before or after the trial depending on the question at hand. Since the Ruckman et al. 2024 decided to extract the post-trial values, we will use that reasoning for not extracting these three additional effect sizes - note that adding these three additional effect sizes would, overall, further reduce the overall effect size.
- **Required action**: None.

## Martin and Hengstebeck 1981

This study appears in the dataset as:

Code

```
Martin_and_Hengstebeck_1981_subset <- metadata.new %>% filter(Authors=="MARTIN, FD; HENGSTEBECK, MF") %>% select(all_of(columns.of.interest))

knitr::kable(Martin_and_Hengstebeck_1981_subset,format = "html")
```

| Authors | Publication.Year | Study | Species | Sample.Size | Stat.Test | Test.Statistic | df1 | df2 | r | n1 | mean1 | sd1 | n2 | mean2 | sd2 | yi | vi |
| --- | --- | --- | --- | --- | --- | --- | --- | --- | --- | --- | --- | --- | --- | --- | --- | --- | --- |
| MARTIN, FD; HENGSTEBECK, MF | 1981 | Martin and Hengstebeck 1981 - 2 | Poecilia reticulata | 33 | X2 | 21.30 | 6 | NA | NA | NA | NA | NA | NA | NA | NA | 0.5680909 | 0.0143343 |
| MARTIN, FD; HENGSTEBECK, MF | 1981 | Martin and Hengstebeck 1981 - 3 | Poecilia reticulata | 12 | X2 | 21.30 | 6 | NA | NA | NA | NA | NA | NA | NA | NA | 0.9420722 | 0.0011506 |
| MARTIN, FD; HENGSTEBECK, MF | 1981 | Martin and Hengstebeck 1981 - 4 | Poecilia reticulata | 61 | X2 | 21.30 | 6 | NA | NA | NA | NA | NA | NA | NA | NA | 0.4178399 | 0.0113550 |
| MARTIN, FD; HENGSTEBECK, MF | 1981 | Martin and Hengstebeck 1981 - 5 | Poecilia reticulata | 64 | X2 | 21.30 | 6 | NA | NA | NA | NA | NA | NA | NA | NA | 0.4079292 | 0.0110298 |
| MARTIN, FD; HENGSTEBECK, MF | 1981 | Martin and Hengstebeck 1981 - 6 | Poecilia reticulata | 27 | X2 | 18.18 | 6 | NA | NA | NA | NA | NA | NA | NA | NA | 0.5802298 | 0.0169235 |
| MARTIN, FD; HENGSTEBECK, MF | 1981 | Martin and Hengstebeck 1981 - 7 | Poecilia reticulata | 11 | X2 | 18.18 | 6 | NA | NA | NA | NA | NA | NA | NA | NA | 0.9090455 | 0.0030150 |
| MARTIN, FD; HENGSTEBECK, MF | 1981 | Martin and Hengstebeck 1981 - 8 | Poecilia reticulata | 16 | X2 | 18.18 | 6 | NA | NA | NA | NA | NA | NA | NA | NA | 0.7537407 | 0.0124344 |
| MARTIN, FD; HENGSTEBECK, MF | 1981 | Martin and Hengstebeck 1981 - 9 | Poecilia reticulata | 11 | X2 | 18.18 | 6 | NA | NA | NA | NA | NA | NA | NA | NA | 0.9090455 | 0.0030150 |
| MARTIN, FD; HENGSTEBECK, MF | 1981 | Martin and Hengstebeck 1981 - 1 | Poecilia reticulata | 74 | F | 3.80 | 2 | 17 | NA | NA | NA | NA | NA | NA | NA | 0.5558265 | 0.0065419 |

For this species (i.e., Poecilia reticulata), Ruckman et al. (2024) classified *Less black eye* as the “Light Color” and *Darker eye* as the “Dark Color”. Nine effect sizes were extracted.

The extracted *F* value corresponds to the following: “*Mean bout lengths of aggressive encounters were 10.3 s for dark-eyed fish, 7.2 s for intermediate fish, and 1.8 s for light-eyed fish (F2,17 = 3.80, P < 0.005)*”.

- **Our assessment**: We think that the extracted *F* value is comparable to those extracted from other studies, and the results suggest that dark-eye fish spend more time on aggressive encounters than light-eyed fish. However, the sample size assigned (74) is not correct based on df2 (17). 74 seems to be the sets of observations performed not the number of individuals.
- **Required action**: Change sample size to 17+2 = 19.

Code

```
metadata.new.updated[metadata.new.updated$Study=="Martin and Hengstebeck 1981 - 1","Sample.Size"] <- 17+2

yi.tmp <- metadata.new.updated[metadata.new.updated$Study=="Martin and Hengstebeck 1981 - 1","yi"]
Sample.Size.tmp <- metadata.new.updated[metadata.new.updated$Study=="Martin and Hengstebeck 1981 - 1","Sample.Size"]

metadata.new.updated[metadata.new.updated$Study=="Martin and Hengstebeck 1981 - 1","vi"] <- ((1-(yi.tmp^2))^2)/(Sample.Size.tmp-1)
```

The 8 extracted *X2* values corresponds to Table IV, where the provided values do not correspond to number of individuals but to number of encounters.

- **Our assessment**: The study does not provide the number of individuals observed to generate the data presented in Table IV, not even an approximate number. The only information on sample sizes is: “*Litters selected for observations had a minimum number of five fish, and for Indiana fish the observed maximum was 21. Some of the Puerto Rico fish were removed on the first day after birth so that the maximum number in a tank was 11*”, but the number of tanks is not reported. For all we can see, all the observations could come from an extremely low number of individuals (even 3, if one would go to the extreme).
- **Required action**: We do not think the *X2* values can be reliably use for the meta-analysis as the sample size is unknown and the raw data not present, and therefore, we are excluding them from the dataset.

Code

```
#saving the useful entry
Martin_and_Hengstebeck_1981.tmp <- metadata.new.updated[metadata.new.updated$Study=="Martin and Hengstebeck 1981 - 1",]

#deleting the rest
metadata.new.updated <- metadata.new.updated[metadata.new.updated$Authors!="MARTIN, FD; HENGSTEBECK, MF",]

# adding the study back
metadata.new.updated <- rbind(metadata.new.updated,Martin_and_Hengstebeck_1981.tmp)
```

## Dijkstra et al 2009b

This study appears in the dataset as:

Code

```
Dijkstra_et_al_2009b_subset <- metadata.new %>% filter(Authors=="Dijkstra, PD; van Dijk, S; Groothuis, TGG; Pierotti, MER; Seehausen, O") %>% select(all_of(columns.of.interest))

knitr::kable(Dijkstra_et_al_2009b_subset,format = "html")
```

| Authors | Publication.Year | Study | Species | Sample.Size | Stat.Test | Test.Statistic | df1 | df2 | r | n1 | mean1 | sd1 | n2 | mean2 | sd2 | yi | vi |
| --- | --- | --- | --- | --- | --- | --- | --- | --- | --- | --- | --- | --- | --- | --- | --- | --- | --- |
| Dijkstra, PD; van Dijk, S; Groothuis, TGG; Pierotti, MER; Seehausen, O | 2009 | Dijkstra et al 2009b | Haplochromis omnicaeruleus | 12 | X2 | 9.5 | 2 | NA | NA | NA | NA | NA | NA | NA | NA | 0.8897565 | 0.0039457 |

For this species (i.e., Haplochromis omnicaeruleus), Ruckman et al. (2024) classified *Plain (orange)*as the “Light Color” and *Black* as the “Dark Color”. Note that Ruckman et al. (2024) wrote that aggression between different morphs were said to be excluded “we therefore limit our data set to measure of aggression within color morphs”. One effect size was extracted.

The extracted *X2* value corresponds to: “*The female morphs differed significantly in ranking (ranking mean+-SE: OB female 1.9 +/- 0.2; P female 2.6 +/-0.5; WB female 1.5 +/- 0.2, Friedman test, X2 = 9.50, df = 2, P = 0.009, n = 12)*”

- **Our assessment**: The authors of the original study report: “*3 distinct female color morphs coexist, black-and-white blotched (WB), orange blotched (OB), and plain (P) color morphs. First, we investigated dominance relationships among female morphs using triadic and dyadic encounters in the laboratory*”. We assume therefore assume that the three morphs are part of a continuum with the extremes being P (plain) and WB (black-and-white), and orange blotched (OB) being intermediate. As far as we can see everything seems correct with the data extraction from this study.
- **Required action**: None.

## Yang et al 2018

This study appears in the dataset as:

Code

```
Yang_et_al_2018_subset <- metadata.new %>% filter(Authors=="Yang, Y; Dugas, MB; Sudekum, HJ; Murphy, SN; Richards-Zawacki, CL") %>% select(all_of(columns.of.interest))

knitr::kable(Yang_et_al_2018_subset,format = "html")
```

| Authors | Publication.Year | Study | Species | Sample.Size | Stat.Test | Test.Statistic | df1 | df2 | r | n1 | mean1 | sd1 | n2 | mean2 | sd2 | yi | vi |
| --- | --- | --- | --- | --- | --- | --- | --- | --- | --- | --- | --- | --- | --- | --- | --- | --- | --- |
| Yang, Y; Dugas, MB; Sudekum, HJ; Murphy, SN; Richards-Zawacki, CL | 2018 | Yang et al 2018 - 1 | Oophaga pumilio | 140 | X2 | 2.10 | 2 | NA | NA | NA | NA | NA | NA | NA | NA | 0.1224745 | 0.0069800 |
| Yang, Y; Dugas, MB; Sudekum, HJ; Murphy, SN; Richards-Zawacki, CL | 2018 | Yang et al 2018 - 2 | Oophaga pumilio | 139 | X2 | 4.25 | 2 | NA | NA | NA | NA | NA | NA | NA | NA | 0.1748586 | 0.0068100 |
| Yang, Y; Dugas, MB; Sudekum, HJ; Murphy, SN; Richards-Zawacki, CL | 2018 | Yang et al 2018 - 3 | Oophaga pumilio | 140 | X2 | 2.77 | 2 | NA | NA | NA | NA | NA | NA | NA | NA | 0.1406617 | 0.0069124 |
| Yang, Y; Dugas, MB; Sudekum, HJ; Murphy, SN; Richards-Zawacki, CL | 2018 | Yang et al 2018 - 4 | Oophaga pumilio | 139 | X2 | 1.77 | 2 | NA | NA | NA | NA | NA | NA | NA | NA | 0.1128442 | 0.0070630 |
| Yang, Y; Dugas, MB; Sudekum, HJ; Murphy, SN; Richards-Zawacki, CL | 2018 | Yang et al 2018 - 5 | Oophaga pumilio | 140 | X2 | 4.16 | 2 | NA | NA | NA | NA | NA | NA | NA | NA | 0.1723783 | 0.0067731 |
| Yang, Y; Dugas, MB; Sudekum, HJ; Murphy, SN; Richards-Zawacki, CL | 2018 | Yang et al 2018 - 6 | Oophaga pumilio | 139 | X2 | 0.61 | 2 | NA | NA | NA | NA | NA | NA | NA | NA | 0.0662457 | 0.0071829 |
| Yang, Y; Dugas, MB; Sudekum, HJ; Murphy, SN; Richards-Zawacki, CL | 2018 | Yang et al 2018 - 7 | Oophaga pumilio | 140 | X2 | 2.33 | 2 | NA | NA | NA | NA | NA | NA | NA | NA | 0.1290072 | 0.0069568 |
| Yang, Y; Dugas, MB; Sudekum, HJ; Murphy, SN; Richards-Zawacki, CL | 2018 | Yang et al 2018 - 8 | Oophaga pumilio | 139 | X2 | 3.16 | 2 | NA | NA | NA | NA | NA | NA | NA | NA | 0.1507774 | 0.0069206 |

For this species (i.e., Oophaga pumilio), Ruckman et al. (2024) classified *Light red*, *Green* and *Red* as the “Light Color” and *Dark red*, *Red* and *Blue* as the “Dark Color”, respectively. Note that Ruckman et al. (2024) wrote that aggression between different morphs were said to be excluded “we therefore limit our data set to measure of aggression within color morphs”. Eight effect sizes were extracted.

The extracted *X2* values corresponds to Table S4-S7, where Likelihood Ratio (LR) X2 are presented: “*The four tables below are generalized linear models evaluating the influence of male color (red, intermediate and blue), model intruder color (red, blue) and their interaction term on the likelihood of a territorial male to track (Table S4), approach (Table S5), call (Table S6) and challenge (Table S7) in the two polymorphic populations. Perch height and conspecific interaction (y/n) were included as covariates*”

- **Our assessment**: The direction of the provided *X2* values in Tables S4-S7 (as well as the general ones provided in Tables 2 and S3) is not provided in the original study. The authors of the original study only provide a direction of the effect for those that are statistically significant, e.g. “*When considering all territorial males, regardless of interaction with conspecifics during the trial, neither the main effects of male colour and intruder colour nor their interaction was a significant predictor of the probability of attack in the high-red polymorphic population (Table 2).*” or “*GLMs for the other four variables (likelihood to track, approach, call and challenge) are presented in Tables S4–S7. We did not detect any significant main effects or an interaction between male colour and model intruder colour in any of the models*”. The only indication for Bluer males being more aggressive than Redder males come from the high-blue polymorphic population in Table S3, where the authors of the original study reported “*However, the likelihood of attack was positively correlated with PC2 (a hue indicator that increases with male ‘blueness’; Table S1), suggesting that bluer males were more aggressive than redder males*”. Table S3 shows PC1 (*X2* = 0.45, p-value = 0.505) and PC2 (*X2* = 6.49, p-value = 0.011), which are quantitative measures as opposed to the “by-eye male colour” categorizations presented in Tables 2 and S4-S7 (Table S10 shows similar results to Table S3 but for “*the subset of observations in which the focal male did not interact with a conspecific*”). According to the authors of the original study, “*PC1 captures the brightness (but much higher green and blue loading) of the male dorsum; PC2 captures hue, or how blue the male was along the red-blue spectrum*”. Summarizing, from the reported results, it is not possible to know the direction of any reported *X2* value other than PC2 in Table S3 (*X2* = 6.49, p-value = 0.011) and the corresponding one in Tables S9 and S10, which present a subset of the same data used in Tables 2 and S3. Thus, without additional information, we cannot assume all those *X2* values are positive.
- **Required action**: Exclude the study. For 7 out of 8 *X2* we do not know the direction of the effect. The only *X2* value for which we know the direction (PC2: *X2* = 6.49, p-value = 0.011, Table S3) is the one corresponding to PC2 in Table S3 - however the corresponding PC1 (which reflects male brightness) is statistically nonsignificant and we do not know in which direction. Thus we think that extracting the only *X2* value for which we know the direction would lead to a biased representation of the findings of the study. Last, all *X2* values provided come from Binomial GLMs rather than *X2* tests, adding additional complexity to their transformation into an effect size.

Code

```
#deleting the study
metadata.new.updated <- metadata.new.updated[metadata.new.updated$Authors!="Yang, Y; Dugas, MB; Sudekum, HJ; Murphy, SN; Richards-Zawacki, CL",]
```

## Rose and Soole 2020

This study appears in the dataset as:

Code

```
Rose_and_Soole_2020_subset <- metadata.new %>% filter(Authors=="Rose, P; Soole, L") %>% select(all_of(columns.of.interest))

knitr::kable(Rose_and_Soole_2020_subset,format = "html")
```

| Authors | Publication.Year | Study | Species | Sample.Size | Stat.Test | Test.Statistic | df1 | df2 | r | n1 | mean1 | sd1 | n2 | mean2 | sd2 | yi | vi |
| --- | --- | --- | --- | --- | --- | --- | --- | --- | --- | --- | --- | --- | --- | --- | --- | --- | --- |
| Rose, P; Soole, L | 2020 | Rose and Soole 2020 | Phoenicoparrus minor | 45 | F | 6.45 | 4 | 40 | 0.33 | NA | NA | NA | NA | NA | NA | 0.6261767 | 0.0083988 |

For this species (i.e., Phoenicoparrus minor), Ruckman et al. (2024) classified *White* as the “Light Color” and *Pink* as the “Dark Color”. One effect size was extracted.

The extracted *F* value corresponds to: “*Differences in time spent on aggression and plumage colour score are significant between birds (F4,40 = 6.45; r2 = 33%; p = .0004).*”

- **Our assessment**: In this study, plumage colour is scored in four categories: 1 being white, and 2 being pink, and from the results shown in Figure 7, from which the *F* value was extracted, it is clear that, as reported in the original study: “*Figure 7 shows that the brightest flamingos are least likely to be seen foraging and being aggressive regardless of the type of foraging location. Birds with a colour score of 3 were most often seen being aggressive; birds with a colour score of 4 had the lowest foraging occurrences*”. Thus, extracting the *F* value, which corresponds to an omnibus test on all four categories would be rather misleading. Instead, the most straightforward way of extracting this result would have been directly from the figure. However, sample sizes for each category are missing. Thus, the second best choice here would be to extract the *t* value, which despite seemingly coming from a GLM, explicitly shows: “*Birds with a brighter plumage are more likely to be aggressive during foraging than paler birds (estimate = 10.23; SE = 4.88; t value = 2.09; p = .04).*”
- **Required action**: To substitute the extracted *F* value by the *t* value, delete the extracted *r* value, which corresponds to the *R2* value of the *F* value, and recalculate the corresponding *r* and *Vr* values using `escalc()` as done for the other *t* values.

Code

```
# making changes 
metadata.new.updated[metadata.new.updated$Study=="Rose and Soole 2020","Stat.Test"] <- "t"
metadata.new.updated[metadata.new.updated$Study=="Rose and Soole 2020","Test.Statistic"] <- 2.09 # no sign change needed
metadata.new.updated[metadata.new.updated$Study=="Rose and Soole 2020","df1"] <- NA
metadata.new.updated[metadata.new.updated$Study=="Rose and Soole 2020","df2"] <- NA
metadata.new.updated[metadata.new.updated$Study=="Rose and Soole 2020","p.value"] <- 0.04
metadata.new.updated[metadata.new.updated$Study=="Rose and Soole 2020","r"] <- NA

# adding the corresponding yi and vi values
metadata.new.updated[metadata.new.updated$Study=="Rose and Soole 2020","yi"] <- escalc(measure = "COR",
                                                                                       ti = 2.09,
                                                                                       ni = 45)[1]
metadata.new.updated[metadata.new.updated$Study=="Rose and Soole 2020","vi"] <- escalc(measure = "COR",
                                                                                       ti = 2.09,
                                                                                       ni = 45)[2]
```

## Podberscek and Serpell 1996

This study appears in the dataset as:

Code

```
Podberscek_and_Serpell_1996_subset <- metadata.new %>% filter(Authors=="Podberscek, AL; Serpell, JA") %>% select(all_of(columns.of.interest))

knitr::kable(Podberscek_and_Serpell_1996_subset,format = "html")
```

| Authors | Publication.Year | Study | Species | Sample.Size | Stat.Test | Test.Statistic | df1 | df2 | r | n1 | mean1 | sd1 | n2 | mean2 | sd2 | yi | vi |
| --- | --- | --- | --- | --- | --- | --- | --- | --- | --- | --- | --- | --- | --- | --- | --- | --- | --- |
| Podberscek, AL; Serpell, JA | 1996 | Podberscek and Serpell 1996 - 1 | Canis lupus familiaris | 428 | t | 2.582 | 427 | NA | NA | NA | NA | NA | NA | NA | NA | 0.1241308 | 0.0022703 |
| Podberscek, AL; Serpell, JA | 1996 | Podberscek and Serpell 1996 - 10 | Canis lupus familiaris | 423 | t | 2.400 | 422 | NA | NA | NA | NA | NA | NA | NA | NA | 0.1161768 | 0.0023061 |
| Podberscek, AL; Serpell, JA | 1996 | Podberscek and Serpell 1996 - 11 | Canis lupus familiaris | 426 | t | 3.492 | 425 | NA | NA | NA | NA | NA | NA | NA | NA | 0.1671993 | 0.0022232 |
| Podberscek, AL; Serpell, JA | 1996 | Podberscek and Serpell 1996 - 12 | Canis lupus familiaris | 428 | t | 3.643 | 427 | NA | NA | NA | NA | NA | NA | NA | NA | 0.1738172 | 0.0022025 |
| Podberscek, AL; Serpell, JA | 1996 | Podberscek and Serpell 1996 - 3 | Canis lupus familiaris | 426 | t | 2.774 | 425 | NA | NA | NA | NA | NA | NA | NA | NA | 0.1335113 | 0.0022698 |
| Podberscek, AL; Serpell, JA | 1996 | Podberscek and Serpell 1996 - 4 | Canis lupus familiaris | 142 | t | 3.365 | 141 | NA | NA | NA | NA | NA | NA | NA | NA | 0.2735472 | 0.0060705 |
| Podberscek, AL; Serpell, JA | 1996 | Podberscek and Serpell 1996 - 6 | Canis lupus familiaris | 425 | t | 3.336 | 424 | NA | NA | NA | NA | NA | NA | NA | NA | 0.1601094 | 0.0022391 |
| Podberscek, AL; Serpell, JA | 1996 | Podberscek and Serpell 1996 - 7 | Canis lupus familiaris | 428 | t | 4.988 | 427 | NA | NA | NA | NA | NA | NA | NA | NA | 0.2349070 | 0.0020906 |
| Podberscek, AL; Serpell, JA | 1996 | Podberscek and Serpell 1996 - 8 | Canis lupus familiaris | 427 | t | 4.524 | 426 | NA | NA | NA | NA | NA | NA | NA | NA | 0.2143458 | 0.0021367 |
| Podberscek, AL; Serpell, JA | 1996 | Podberscek and Serpell 1996 - 9 | Canis lupus familiaris | 428 | t | 3.161 | 427 | NA | NA | NA | NA | NA | NA | NA | NA | 0.1513858 | 0.0022358 |

For this species (i.e., Canis lupus familiaris), Ruckman et al. (2024) classified *Black* as the “Light Color” and *Red/golden* as the “Dark Color”. Ten effect sizes were extracted (83% of all mammal ones, 10/12).

Results are shown in: “*Within the solid colour group, red/goldens were compared with blacks. Here it was found that red/goldens were significantly more likely to be aggressive in a number of situations. These included, Al (towards strange dogs; Mann-Whitney U test, Z = 2.582, P < 0.01), A4 (towards persons approaching owner away from home; Z = 2.774, P < 0.011, A5 (towards children in the household; Z= 3.365, P < 0.001), A7 (when owner gives attention to other person or animal; Z = 3.336, P < 0.001), A8 (toward owner or member of owner’s family; Z= 4.988, P < 0.001), A9 (when disciplined; Z= 4.524, P < 0.001)>, A10 (when reached for or handled; Z= 3.161, P < 0.011, All (when in restricted spaces; Z = 2.4, P < 0.05>, Al2 (at meal times/ defending food; Z = 3.492, P < 0.001)), Al3 (sudden and without apparent reason; Z= 3.643, P < 0.001).*”

- **Our assessment**: Ruckman et al. 2024 established the following criterium: “*We defined aggression as any variable that measured antagonistic behaviors (e.g., biting or chasing) toward a conspecific (of same sex, color class, and age class) or mirror image.*” Of the 13 questions asked of the dogs’ owners in Podberscek and Serpell 1996, only two (A1 and A6) refer to aggression towards conspecifics (A1 and A6), see table 1 of the original paper. Hence, only these two should be considered. Of these 2, only 1 (A1) is significant and therefore reported, as only statistically significant findings were reported. Only extracting significant results would bias the results, and therefore, even this single effect size should be excluded. Moreover, the methodology used (dog owner surveys) is not at all comparable with the rest of the studies where aggression was measured directly, and therefore, we think that the study should have been excluded a priori in any case.
- **Required action**: Exclude this study.

Code

```
metadata.new.updated <- metadata.new.updated[metadata.new.updated$Authors!="Podberscek, AL; Serpell, JA",]
```

## Karlsson et al. 2011

This study appears in the dataset as:

Code

```
Karlsson_et_al_2011_subset <- metadata.new %>% filter(Authors=="Karlsson, AC; Mormede, P; Kerje, S; Jensen, P") %>% select(all_of(columns.of.interest))

knitr::kable(Karlsson_et_al_2011_subset,format = "html")
```

| Authors | Publication.Year | Study | Species | Sample.Size | Stat.Test | Test.Statistic | df1 | df2 | r | n1 | mean1 | sd1 | n2 | mean2 | sd2 | yi | vi |
| --- | --- | --- | --- | --- | --- | --- | --- | --- | --- | --- | --- | --- | --- | --- | --- | --- | --- |
| Karlsson, AC; Mormede, P; Kerje, S; Jensen, P | 2011 | Karlsson et al 2011 - 1 | Gallus gallus domesticus | 23 | mean | NA | NA | NA | NA | 9 | 2.2 | 1.500 | 14 | 1.1 | 1.1225 | -0.5109224 | 0.0464459 |
| Karlsson, AC; Mormede, P; Kerje, S; Jensen, P | 2011 | Karlsson et al 2011 - 2 | Gallus gallus domesticus | 22 | mean | NA | NA | NA | NA | 6 | 2.0 | 1.715 | 16 | 3.6 | 2.4000 | 0.4227407 | 0.0643280 |
| Karlsson, AC; Mormede, P; Kerje, S; Jensen, P | 2011 | Karlsson et al 2011 - 3 | Gallus gallus domesticus | 23 | mean | NA | NA | NA | NA | 9 | 1.5 | 1.800 | 14 | 2.0 | 2.6192 | 0.1378032 | 0.0712365 |
| Karlsson, AC; Mormede, P; Kerje, S; Jensen, P | 2011 | Karlsson et al 2011 - 4 | Gallus gallus domesticus | 22 | mean | NA | NA | NA | NA | 6 | 0.0 | 0.000 | 16 | 6.2 | 8.0000 | 0.5168769 | 0.0549581 |

For this species (i.e., Gallus gallus domesticus), Ruckman et al. (2024) classified *White* as the “Light Color” and *Wild type (red)* as the “Dark Color”. Note that Ruckman et al. (2024) wrote that aggression between different morphs were said to be excluded “we therefore limit our data set to measure of aggression within color morphs”. Four effect sizes were extracted.

Results are shown in Table 2.

- **Our assessment**: All values were extracted correctly.
- **Required action**: None.

## Seaver and Hurd 2017

This study appears in the dataset as:

Code

```
Seaver_and_Hurd_2017_subset <- metadata.new %>% filter(Authors=="Seaver, CMS; Hurd, PL") %>% select(all_of(columns.of.interest))

knitr::kable(Seaver_and_Hurd_2017_subset,format = "html")
```

| Authors | Publication.Year | Study | Species | Sample.Size | Stat.Test | Test.Statistic | df1 | df2 | r | n1 | mean1 | sd1 | n2 | mean2 | sd2 | yi | vi |
| --- | --- | --- | --- | --- | --- | --- | --- | --- | --- | --- | --- | --- | --- | --- | --- | --- | --- |
| Seaver, CMS; Hurd, PL | 2017 | Seaver and Hurd 2017 - 1 | Pelvicachromis pulcher | 85 | mean | NA | NA | NA | NA | 70 | 6.703 | 1.058 | 15 | 9.937 | 3.293 | 0.8834182 | 0.0055856 |
| Seaver, CMS; Hurd, PL | 2017 | Seaver and Hurd 2017 - 2 | Pelvicachromis pulcher | 85 | mean | NA | NA | NA | NA | 70 | 3.468 | 0.803 | 15 | 1.783 | 0.431 | -0.9611099 | 0.0034565 |
| Seaver, CMS; Hurd, PL | 2017 | Seaver and Hurd 2017 - 3 | Pelvicachromis pulcher | 85 | mean | NA | NA | NA | NA | 70 | 4.645 | 0.941 | 15 | 9.761 | 3.470 | 1.1248359 | 0.0025228 |
| Seaver, CMS; Hurd, PL | 2017 | Seaver and Hurd 2017 - 4 | Pelvicachromis pulcher | 85 | mean | NA | NA | NA | NA | 70 | 325.038 | 42.573 | 15 | 457.507 | 135.023 | 0.8863804 | 0.0054991 |

For this species (i.e., Pelvicachromis pulcher), Ruckman et al. (2024) classified *Yellow* as the “Light Color” and *Red* as the “Dark Color”. Note that Ruckman et al. (2024) wrote that aggression between different morphs were said to be excluded “we therefore limit our data set to measure of aggression within color morphs”. Four effect sizes were extracted.

The effects sizes were seemingly extracted from Figure 2.

- **Our assessment**: All values were extracted correctly. However, there seem to be an additional effect size that could have been extracted: “*There was no significant difference between females, yellow males and red males in the proportion that showed aggression to their mirror image (X22 = 3.20, p = 0.20; Table 1)*”. The corresponding 2x2 contingency table for that result would be:

Code

```
#create table
table1.Seaver <- matrix(c(19,51,4,11), ncol=2, byrow=TRUE)
rownames(table1.Seaver) <- c("yellow","red")
colnames(table1.Seaver) <- c("nonaggressor","aggressor")
table1.Seaver <- as.table(table1.Seaver)
table1.Seaver
```

```
       nonaggressor aggressor
yellow           19        51
red               4        11
```

From which we can calculate the corresponding X2 value as:

Code

```
#chisq.test(table1.Seaver)
chisq.test(table1.Seaver,correct=F)
```

```
Warning in chisq.test(table1.Seaver, correct = F): Chi-squared approximation
may be incorrect
```

```
    Pearson's Chi-squared test

data:  table1.Seaver
X-squared = 0.0014192, df = 1, p-value = 0.9699
```

- **Required action**: To add this additional effect size by transforming the X2 value to an *r* value following the procedures used for the other studies.

Code

```
Seaver_and_Hurd_2017_extra_1 <- metadata.new %>% filter(Study=="Seaver and Hurd 2017 - 1")

# emptying entry
Seaver_and_Hurd_2017_extra_1[,c(1:ncol(Seaver_and_Hurd_2017_extra_1))] <- NA


# adding variables of interest from original sources
Seaver_and_Hurd_2017_extra_1[,c("Authors","Publication.Year","Species")] <- Seaver_and_Hurd_2017_subset[1,c("Authors","Publication.Year","Species")]

Seaver_and_Hurd_2017_extra_1[,"Study"] <- "Seaver and Hurd 2017 - 5"
Seaver_and_Hurd_2017_extra_1[,"Sample.Size"] <- sum(table1.Seaver)
Seaver_and_Hurd_2017_extra_1[,"Stat.Test"] <- "X2"
Seaver_and_Hurd_2017_extra_1[,"Test.Statistic"] <- chisq.test(table1.Seaver,correct=F)$statistic[[1]]
```

```
Warning in chisq.test(table1.Seaver, correct = F): Chi-squared approximation
may be incorrect
```

Code

```
Seaver_and_Hurd_2017_extra_1[,"df1"] <- chisq.test(table1.Seaver,correct=F)$parameter[[1]]
```

```
Warning in chisq.test(table1.Seaver, correct = F): Chi-squared approximation
may be incorrect
```

Code

```
Seaver_and_Hurd_2017_extra_1[,"p.value"] <- chisq.test(table1.Seaver,correct=F)$p.value[[1]]
```

```
Warning in chisq.test(table1.Seaver, correct = F): Chi-squared approximation
may be incorrect
```

Code

```
# caculating corresponding r
Seaver_and_Hurd_2017_extra_1[,"yi"] <- sqrt((chisq.test(table1.Seaver,correct=F)$statistic[[1]])/
                                              (sum(table1.Seaver)*(nrow(table1.Seaver)-1)))
```

```
Warning in chisq.test(table1.Seaver, correct = F): Chi-squared approximation
may be incorrect
```

Code

```
# calculating vi
Seaver_and_Hurd_2017_extra_1[,"vi"] <- ((1 - (Seaver_and_Hurd_2017_extra_1[,"yi"] ^ 2)) ^ 2)/(sum(table1.Seaver) - 1)

# finally, adding this entry to the new dataset
metadata.new.updated <- rbind(metadata.new.updated,
                              Seaver_and_Hurd_2017_extra_1)
```

## Zinzow-Kramer et al. 2015

This study appears in the dataset as:

Code

```
Zinzow_Kramer_et_al_2015_subset <- metadata.new %>% filter(Authors=="Zinzow-Kramer, WM; Horton, BM; McKee, CD; Michaud, JM; Tharp, GK; Thomas, JW; Tuttle, EM; Yi, S; Maney, DL") %>% select(all_of(columns.of.interest))

knitr::kable(Zinzow_Kramer_et_al_2015_subset,format = "html")
```

| Authors | Publication.Year | Study | Species | Sample.Size | Stat.Test | Test.Statistic | df1 | df2 | r | n1 | mean1 | sd1 | n2 | mean2 | sd2 | yi | vi |
| --- | --- | --- | --- | --- | --- | --- | --- | --- | --- | --- | --- | --- | --- | --- | --- | --- | --- |
| Zinzow-Kramer, WM; Horton, BM; McKee, CD; Michaud, JM; Tharp, GK; Thomas, JW; Tuttle, EM; Yi, S; Maney, DL | 2015 | Zinzow-Kramer et al 2015 - 1 | Zonotrichia albicollis | 19 | mean | NA | NA | NA | NA | 10 | 23.516 | 19.6829630 | 9 | 1.733 | 7.0893827 | -0.7591132 | 0.0257579 |
| Zinzow-Kramer, WM; Horton, BM; McKee, CD; Michaud, JM; Tharp, GK; Thomas, JW; Tuttle, EM; Yi, S; Maney, DL | 2015 | Zinzow-Kramer et al 2015 - 2 | Zonotrichia albicollis | 19 | mean | NA | NA | NA | NA | 10 | 0.000 | 0.2469136 | 9 | 0.000 | 0.4938272 | 0.0000000 | 0.0874048 |
| Zinzow-Kramer, WM; Horton, BM; McKee, CD; Michaud, JM; Tharp, GK; Thomas, JW; Tuttle, EM; Yi, S; Maney, DL | 2015 | Zinzow-Kramer et al 2015 - 3 | Zonotrichia albicollis | 19 | mean | NA | NA | NA | NA | 10 | 20.846 | 41.3876543 | 9 | 16.172 | 19.3279012 | -0.0937890 | 0.0861864 |
| Zinzow-Kramer, WM; Horton, BM; McKee, CD; Michaud, JM; Tharp, GK; Thomas, JW; Tuttle, EM; Yi, S; Maney, DL | 2015 | Zinzow-Kramer et al 2015 - 4 | Zonotrichia albicollis | 19 | mean | NA | NA | NA | NA | 10 | 7.565 | 9.0933333 | 9 | 11.342 | 11.4419753 | 0.2391339 | 0.0796381 |
| Zinzow-Kramer, WM; Horton, BM; McKee, CD; Michaud, JM; Tharp, GK; Thomas, JW; Tuttle, EM; Yi, S; Maney, DL | 2015 | Zinzow-Kramer et al 2015 - 5 | Zonotrichia albicollis | 19 | mean | NA | NA | NA | NA | 10 | 10.154 | 0.3037037 | 9 | 10.155 | 0.8355556 | 0.0010778 | 0.0874046 |
| Zinzow-Kramer, WM; Horton, BM; McKee, CD; Michaud, JM; Tharp, GK; Thomas, JW; Tuttle, EM; Yi, S; Maney, DL | 2015 | Zinzow-Kramer et al 2015 - 6 | Zonotrichia albicollis | 19 | mean | NA | NA | NA | NA | 10 | 7.168 | 4.4069136 | 9 | 6.047 | 4.0217284 | -0.1737715 | 0.0832583 |
| Zinzow-Kramer, WM; Horton, BM; McKee, CD; Michaud, JM; Tharp, GK; Thomas, JW; Tuttle, EM; Yi, S; Maney, DL | 2015 | Zinzow-Kramer et al 2015 - 7 | Zonotrichia albicollis | 19 | mean | NA | NA | NA | NA | 10 | 0.782 | 0.6508642 | 9 | 0.977 | 1.0834568 | 0.1455103 | 0.0844867 |
| Zinzow-Kramer, WM; Horton, BM; McKee, CD; Michaud, JM; Tharp, GK; Thomas, JW; Tuttle, EM; Yi, S; Maney, DL | 2015 | Zinzow-Kramer et al 2015 - 8 | Zonotrichia albicollis | 19 | mean | NA | NA | NA | NA | 10 | 0.247 | 0.2128395 | 9 | 0.354 | 0.6306173 | 0.1530315 | 0.0841802 |

For this species (i.e., Zonotrichia albicollis), Ruckman et al. (2024) classified *White (WS)* as the “Light Color” and *Tan (TS)* as the “Dark Color”. Eight effect sizes were extracted.

The effects sizes were extracted from Figure 1.

- **Our assessment**: All values extracted are correct, but there are two effect sizes for which the sign have been flipped. Those two correspond to latency to approach (the time from start of playback until the resident male arrived: the longer, the more scared), and distance of closest approach to the decoy (the further, the more scared). In addition, the extracted values correspond to medians rather than means, which should have probably accounted for since medians can be rather far from means when data is skewed (for more on this, see https://training.cochrane.org/handbook/current/chapter-06#section-6-5-2). We ignore this last issue as likely inconsequential.
- **Required action**: To assign a negative sign to effect sizes corresponding to latency to approach and instance of closest approach to the decoy.

Code

```
# adjusting the sign accordingly 
metadata.new.updated[metadata.new.updated$Study=="Zinzow-Kramer et al 2015 - 7","yi"] <- metadata.new.updated[metadata.new.updated$Study=="Zinzow-Kramer et al 2015 - 7","yi"]*(-1)
metadata.new.updated[metadata.new.updated$Study=="Zinzow-Kramer et al 2015 - 8","yi"] <- metadata.new.updated[metadata.new.updated$Study=="Zinzow-Kramer et al 2015 - 8","yi"]*(-1)
```

# Describing differences between original and updated dataset

After accounting for the issues found in 8 out 11 studies (64%) that were reassessed and that correspond to 15% of all studies included in Ruckman et al. (2024), the **new and updated dataset** contains 147 effect sizes extracted from 72 studies and covering 55 species\*, whereas the **original dataset** contained 169 effect sizes extracted from 74 studies and covering 55 species\*.

\* Note that the final number of species for the analysis is 54 because we renamed *Haplochromis omnicaeruleus* as *Haplochromis paludinosus* following the updated taxonomic information.

Our reassessment allowed us to reduce (but likely not eliminate) the consequences of the excess of positive values found in the original dataset, which, based on our reassessment we believe to be largely caused by an incorrect management of effect size direction (more below). Indeed, whereas the percentage of positive values for each effect size origin for the **original dataset** looked like:

Code

```
################################################################################
# Exploring effect size type disagreements
################################################################################

# calculate percentage of positive values for each type of effect size for
# the original
effect.size.positive.perc.original <- metadata.new %>% 
  group_by(Stat.Test) %>% 
  mutate(Stat.Test = factor(Stat.Test, 
                            levels = c("r","mean","t","F","X2"))) %>%
  mutate(Stat.Test = recode(Stat.Test, r = "Pearson's r",
                            mean = "mean, SD, N",
                            t = "t value",
                            F = "F value",
                            X2 = "X2 value")) %>% 
  summarise(Percentage = round(100*table(yi<0)[1]/n(),1))# %>% 

knitr::kable(effect.size.positive.perc.original,format = "html") # output format specification is optional
```

| Stat.Test | Percentage |
| --- | --- |
| Pearson's r | 44.0 |
| mean, SD, N | 61.0 |
| t value | 85.7 |
| F value | 93.9 |
| X2 value | 100.0 |

The corresponding percentages for the **new and updated** dataset looked like:

Code

```
# and the updated database
effect.size.positive.perc.updated <- metadata.new.updated %>%
  group_by(Stat.Test) %>%
  mutate(Stat.Test = factor(Stat.Test,
                            levels = c("r","mean","t","F","X2"))) %>%
  mutate(Stat.Test = recode(Stat.Test, r = "Pearson's r",
                            mean = "mean, SD, N",
                            t = "t value",
                            F = "F value",
                            X2 = "X2 value")) %>%
  summarise(Percentage = round(100*table(yi<0)[1]/n(),1))# %>% 

knitr::kable(effect.size.positive.perc.updated,format = "html") # output format specification is optional
```

| Stat.Test | Percentage |
| --- | --- |
| Pearson's r | 44.0 |
| mean, SD, N | 54.8 |
| t value | 80.8 |
| F value | 88.2 |
| X2 value | 100.0 |

Here are the corresponding figures for the **original dataset**:

Code

```
# generating the data subset
metadata.original.yi <- metadata.new %>%
  select(c(yi,vi,Stat.Test)) %>%
  mutate(Stat.Test = factor(Stat.Test,
                            levels = c("r","mean","t","F","X2"))) %>%
  mutate(Stat.Test = recode(Stat.Test, r = "Pearson's r",
                            mean = "mean, SD, N",
                            t = "t value",
                            F = "F value",
                            X2 = "X2 value"))

# generating label for annotation
effect.size.positive.perc.original$label.perc <- paste0(round(effect.size.positive.perc.original$Percentage,0),
                                                        "%\npositive")


# effect size magnitude # more at: https://indrajeetpatil.github.io/ggstatsplot/reference/ggbetweenstats.html
set.seed(77)
yi.plot.original <- ggbetweenstats(
  data  = metadata.original.yi,
  x     = Stat.Test,
  y     = yi,
  point.args = list(position = ggplot2::position_jitterdodge(dodge.width = 0.6),
                    alpha = 0.4,
                    size = 1/sqrt(metadata.original.yi$vi)-min(1/sqrt(metadata.original.yi$vi))+0.1,
                    stroke = 0, na.rm = TRUE),
  #point.args = list(size = 1),
  type = "parametric",
  pairwise.display = "none",
  #p.adjust.method = "none", # if no multiple correction used, differences are everywhere
  #ggsignif.args = list(textsize = 3, tip_length = 0.02, na.rm = TRUE), # if pairwise.display on, change size
  bf.message = F,
  effsize.type = "eta", # which corresponds to the partial eta squared we are using to transform F-to-r
  #results.subtitle = F, # to remove statistical results from the top of the plot
  centrality.label.args = list(size = 3, nudge_x = 0.4,
                               segment.linetype = 3,
                               min.segment.length = 0),
  xlab = "\nEffect size origin\n",
  ylab = "\nEffect size\n(r and rbis)",
  title = "\nDoes effect size magnitude differ considerably depending on their origin?"
) +
  # modifying text size
  theme(axis.text=element_text(size=10),
        axis.title=element_text(size=11,face="bold"),
        plot.title = element_text(size=12)) +
  # adding the percentage of positive effect sizes for each type
  annotate("text",
           x = seq(0.65,4.65,1),
           y = 1.1,
           label = effect.size.positive.perc.original$label.perc) +
  # adding grey area to better signal postive vs negative values
  annotate("rect", xmin = 0, xmax = 6, ymin = -1, ymax = 0,
           alpha = .1)

yi.plot.original
```

and the **new and updated dataset**:

Code

```
# generating the data subset
metadata.updated.yi <- metadata.new.updated %>%
  select(c(yi,vi,Stat.Test)) %>%
  mutate(Stat.Test = factor(Stat.Test,
                            levels = c("r","mean","t","F","X2"))) %>%
  mutate(Stat.Test = recode(Stat.Test, r = "Pearson's r",
                            mean = "mean, SD, N",
                            t = "t value",
                            F = "F value",
                            X2 = "X2 value"))

# generating label for annotation
effect.size.positive.perc.updated$label.perc <- paste0(round(effect.size.positive.perc.updated$Percentage,0),
                                                        "%\npositive")


# effect size magnitude # more at: https://indrajeetpatil.github.io/ggstatsplot/reference/ggbetweenstats.html
set.seed(77)
yi.plot.updated <- ggbetweenstats(
  data  = metadata.updated.yi,
  x     = Stat.Test,
  y     = yi,
  point.args = list(position = ggplot2::position_jitterdodge(dodge.width = 0.6),
                    alpha = 0.4,
                    size = 1/sqrt(metadata.updated.yi$vi)-min(1/sqrt(metadata.updated.yi$vi))+0.1,
                    stroke = 0, na.rm = TRUE),
  #point.args = list(size = 1),
  type = "parametric",
  pairwise.display = "none",
  #p.adjust.method = "none", # if no multiple correction used, differences are everywhere
  #ggsignif.args = list(textsize = 3, tip_length = 0.02, na.rm = TRUE), # if pairwise.display on, change size
  bf.message = F,
  effsize.type = "eta", # which corresponds to the partial eta squared we are using to transform F-to-r
  #results.subtitle = F, # to remove statistical results from the top of the plot
  centrality.label.args = list(size = 3, nudge_x = 0.4,
                               segment.linetype = 3,
                               min.segment.length = 0),
  xlab = "\nEffect size origin\n",
  ylab = "\nEffect size\n(r and rbis)",
  title = "\nDoes effect size magnitude differ considerably depending on their origin?"
) +
  # modifying text size
  theme(axis.text=element_text(size=10),
        axis.title=element_text(size=11,face="bold"),
        plot.title = element_text(size=12)) +
  # adding the percentage of positive effect sizes for each type
  annotate("text",
           x = seq(0.65,4.65,1),
           y = 1.1,
           label = effect.size.positive.perc.updated$label.perc) +
  # adding grey area to better signal postive vs negative values
  annotate("rect", xmin = 0, xmax = 6, ymin = -1, ymax = 0,
           alpha = .1)

yi.plot.updated
```

Based on our exploration of 15% of all studies included in Ruckman et al. (2024), the excess of positive values found is likely due to an incorrect assignment of effect size direction in the original dataset due to: (1) not adjusting the direction of effect size of traits for which larger means less aggressive (e.g., latency to approach), (2) assigning a positive sign to directionless inferential statistics such as *F* and *X2* values, and (3) an unexpected lower likelihood of negative effect sizes.

# Conclusions

Based on our reassessment of 15% of all studies included in Ruckman et al. (2024) we cannot guarantee the reliability of the dataset. That is, despite that we have fixed the issues found in 73% of all reassessed studies, there is strong evidence suggesting that those (and possibly other) issues will be present for a substantial percentage of the remaining 85% of the studies that we did not reassessed. Thus, our re-analyses should be interpret with extreme caution as there is evidence to expect that their results will still exaggerate the *true* association between aggression and coloration.

The code below saves the *new and updated dataset* for the corresponding analyses.

Code

```
# saving dataset
write.csv(metadata.new.updated, "../data/new/meta_complete_data2_new_and_updated.csv")
```

### R session info

Code

```
sessionInfo()
```

```
R version 4.3.1 (2023-06-16 ucrt)
Platform: x86_64-w64-mingw32/x64 (64-bit)
Running under: Windows 10 x64 (build 19044)

Matrix products: default


locale:
[1] LC_COLLATE=English_Germany.utf8  LC_CTYPE=English_Germany.utf8   
[3] LC_MONETARY=English_Germany.utf8 LC_NUMERIC=C                    
[5] LC_TIME=English_Germany.utf8    

time zone: Europe/London
tzcode source: internal

attached base packages:
[1] stats     graphics  grDevices utils     datasets  methods   base     

other attached packages:
[1] knitr_1.49          ggplot2_3.5.1       dplyr_1.1.4        
[4] ggstatsplot_0.12.5  metafor_4.6-0       numDeriv_2016.8-1.1
[7] metadat_1.2-0       Matrix_1.6-1       

loaded via a namespace (and not attached):
 [1] gtable_0.3.6           xfun_0.49              bayestestR_0.15.0     
 [4] htmlwidgets_1.6.4      insight_1.4.2          ggrepel_0.9.6         
 [7] lattice_0.21-8         paletteer_1.6.0        mathjaxr_1.6-0        
[10] vctrs_0.6.5            tools_4.3.1            generics_0.1.3        
[13] datawizard_1.2.0       sandwich_3.1-0         tibble_3.2.1          
[16] pacman_0.5.1           pkgconfig_2.0.3        correlation_0.8.6     
[19] lifecycle_1.0.4        compiler_4.3.1         farver_2.1.2          
[22] munsell_0.5.1          codetools_0.2-19       htmltools_0.5.8       
[25] yaml_2.3.10            pillar_1.10.1          tidyr_1.3.1           
[28] MASS_7.3-60            statsExpressions_1.6.1 multcomp_1.4-28       
[31] nlme_3.1-162           tidyselect_1.2.1       digest_0.6.35         
[34] mvtnorm_1.3-2          purrr_1.2.1            rematch2_2.1.2        
[37] labeling_0.4.3         splines_4.3.1          fastmap_1.1.1         
[40] grid_4.3.1             colorspace_2.1-0       cli_3.6.1             
[43] magrittr_2.0.3         patchwork_1.3.0        survival_3.5-5        
[46] TH.data_1.1-3          withr_3.0.2            scales_1.3.0          
[49] estimability_1.5.1     rmarkdown_2.29         emmeans_1.10.6        
[52] zoo_1.8-14             coda_0.19-4            evaluate_1.0.3        
[55] parameters_0.23.0      rlang_1.1.4            Rcpp_1.1.0            
[58] zeallot_0.1.0          xtable_1.8-4           glue_1.8.0            
[61] rstudioapi_0.17.1      jsonlite_1.8.8         effectsize_0.8.9      
[64] R6_2.6.1               prismatic_1.1.2
```


##### Source Code

```
---
title: "Supporting Information for: Fifty years later, and we still don’t know about badges of status"
authors: "Alfredo Sanchez-Tojar, Pietro B. D’Amelio"
#css: style.css
format: 
  html:
    toc: true
    toc-location: left
    code-fold: true
    code-tools: true
    code-block-bg: true
    code-block-border-left: "#31BAE9"
    code-copy: hover
    code-overflow: wrap
editor: visual
---

```{r Setup, include=FALSE}
# Clear memory
rm(list=ls()) 

# install.packages("pacman")
pacman::p_load(metafor,
               ggstatsplot,
               dplyr,
               ggplot2,
               knitr)
```

# Importing original new dataset

This corresponds to the dataset containing all recalculated effect sizes generated by script '001_effect_size_calculation.R'.

```{r}
metadata.new <- read.csv("../data/new/meta_complete_data2_new.csv", header=T)

#excluding it here already because the authors decided to exclude from the final analyses
metadata.new <- metadata.new %>%
  filter(Classification!="pteridine")

# creating a copy for fixing and adding effect sizes
metadata.new.updated <- metadata.new

# list of columns of interest for re-extracting and adding new effect sizes
columns.of.interest <- c("Authors","Publication.Year","Study","Species",
                         "Sample.Size","Stat.Test","Test.Statistic","df1","df2",
                         "r","n1","mean1","sd1","n2","mean2","sd2","yi","vi")


knitr::kable(head(metadata.new[,-c(1:2)]),format = "html")
```

<br>

In the following sections, we reassess the data extraction of 11 studies, which correspond to `r round(100*11/length(unique(metadata.new$Authors)),0)`% of all studies included in the meta-analysis of Ruckman et al. (2024).

## Andrews et al. 1996

This study appears in the dataset as:

```{r}
Andrews_et_al_1996_subset <- metadata.new %>% filter(Authors=="Andrews, TJ; Summers, CH") %>% select(all_of(columns.of.interest))

knitr::kable(Andrews_et_al_1996_subset,format = "html")
```

<br>

For this species (i.e., `r unique(Andrews_et_al_1996_subset$Species)`), Ruckman et al. (2024) classified *Light green* as the "Light Color" and *Dark green or Brown* as the "Dark Color". Two effect sizes were extracted.

The *t* value was extracted from following original text: "*Dominant and subordinate females were also not significantly (t16 = 1.92, p \> 0.072) different in mean body color in the absence of a male; all visible pigmented body surface of both females were a light to moderate green*"

-   **Our assessment**: The *t* value comes from an independent *t* test comparing subordinate vs dominant for the no male condition. Performed data extraction is clear.

-   **Required action**: None.

The F value was extracted from following original text: "*However, when males were present body coloration was significantly darker in all females, and statistically darkest in dominant or single females (F~5,53~ = 16.393, p \< 0.001)*"

-   **Our assessment**: The *F* value seems to correspond to the following ANOVA: "*Comparisons were made statistically for aggressive, submissive and courtship behavior, perch site selection and color by paired t-test or ANOVA*"), which contains 2 predictors: (1) context (levels: no male, male present), and treatment (levels: single, subordinate, dominant), which explain why df1 = 5. The reason for df2 = 53 is because there are 9 females in each group for a total of 54. Since 18 rather than 54 is used as the sample size when calculating *Vr*, there is no action required for this study.

-   **Required action**: None.

## Carola et al. 2014

This study appears in the dataset as:

```{r}
Carola_et_al_2014_subset <- metadata.new %>% filter(Authors=="Carola, V; Perlas, E; Zonfrillo, F; Soini, HA; Novotny, MV; Gross, CT") %>% select(all_of(columns.of.interest))

knitr::kable(Carola_et_al_2014_subset,format = "html")
```

<br>

For this species (i.e., `r unique(Carola_et_al_2014_subset$Species)`), Ruckman et al. (2024) classified *Non-agouti* as the "Light Color" and *Agouti* as the "Dark Color". Note that Ruckman et al. (2024) wrote that aggression between different morphs were said to be excluded "we therefore limit our data set to measure of aggression within color morphs". One effect size was extracted.

The F value was extracted from following original text: "*Non-agouti mice showed significantly increased aggressive-like behavior when compared to agouti littermates in the test, exhibiting more attacks \[Figure 1A; repeated measure ANOVA, genotype effect: F~1,18~ = 5.40, P = 0.032\]*"

-   **Our assessment**: Since the Non-agouti is considered the "Light Color", the sign of the final effect size should be negative, which is.

-   **Required action**: None.

When revisiting this study, we realized that there was an additional aggression proxy that was not extracted: "*...and a shorter latency to the first attack \[Figure 1B; repeated measure ANOVA, genotype effect: F~1,18~ = 7.77; P = 0.012\] toward a non-agouti intruder over three consecutive trials.*".

-   **Our assessment**: There is no clear reason why this proxy was not extracted since latency was extracted for other studies in the dataset. In the full dataset there are several other papers where multiple effect sizes from the same group of animals were extracted. This sort of nonindependence (i.e., multiple estimates from the same group of animals) should be accounted for with a random effect (i.e., "Group ID"). This should be done for all such cases in the dataset.

-   **Required action**: We extracted the data for the additional effect size from Figure 1B using the R package `metaDigitise` (Pick et al. 2019). The corresponding *r~bis~* are calculated below and then added to the database.

```{r}
Carola_et_al_2014_extra_1 <- metadata.new %>% filter(Authors=="Carola, V; Perlas, E; Zonfrillo, F; Soini, HA; Novotny, MV; Gross, CT")

# emptying entry
Carola_et_al_2014_extra_1[,c(1:ncol(Carola_et_al_2014_extra_1))] <- NA

# adding variables of interest from original sources
Carola_et_al_2014_extra_1[,c("Authors","Publication.Year","Species")] <- Carola_et_al_2014_subset[,c("Authors","Publication.Year","Species")]

Carola_et_al_2014_extra_1[,"Study"] <- "Carola et al 2014 - 2"
Carola_et_al_2014_extra_1[,"Sample.Size"] <- 20
Carola_et_al_2014_extra_1[,"Stat.Test"] <- "mean"
Carola_et_al_2014_extra_1[,"Test.Statistic"] <- 7.77
Carola_et_al_2014_extra_1[,"df1"] <- 1
Carola_et_al_2014_extra_1[,"df2"] <- 18
Carola_et_al_2014_extra_1[,"n1"] <- 10
Carola_et_al_2014_extra_1[,"mean1"] <- 22.94877
Carola_et_al_2014_extra_1[,"sd1"] <- 10.578670
Carola_et_al_2014_extra_1[,"n2"] <- 10
Carola_et_al_2014_extra_1[,"mean2"] <- 35.74442
Carola_et_al_2014_extra_1[,"sd2"] <- 9.785269

# caculating rbis
Carola_et_al_2014_extra_1 <- as.data.frame(escalc(measure = "RBIS",
                                                  n2i = n1,
                                                  n1i = n2,
                                                  m2i = mean1,
                                                  m1i = mean2,
                                                  sd2i = sd1,
                                                  sd1i = sd2,
                                                  data = Carola_et_al_2014_extra_1))

# flipping the sign to reflect that it is the Non-agouti (Light Color) that takes less time to attack
Carola_et_al_2014_extra_1[,"yi"] <- Carola_et_al_2014_extra_1[,"yi"] * (-1)

# finally, adding this entry to the new dataset
metadata.new.updated <- rbind(metadata.new.updated,Carola_et_al_2014_extra_1)
```

There seem to be several other effect sizes that could have been extracted from this paper: "*To evaluate if aggressive behavior of the resident could be modulated by the genotype of the intruder a fourth trial was carried out in which each group was split and half were exposed to non-agouti and the other half to agouti intruders. No significant behavioral differences between mice exposed to agouti or non-agouti intruders were detected (Figure S2)*".

## Naretto and Chiaraviglio 2023

This study appears in the dataset as:

```{r}
Naretto_and_Chiaraviglio_2023_subset <- metadata.new %>% filter(Authors=="Naretto, Sergio; Chiaraviglio, Margarita") %>% select(all_of(columns.of.interest))

knitr::kable(Naretto_and_Chiaraviglio_2023_subset,format = "html")
```

<br>

For this species (i.e., `r unique(Naretto_and_Chiaraviglio_2023_subset$Species)`), Ruckman et al. (2024) classified *Lighter* as the "Light Color" and *Darker* as the "Dark Color". One effect size was extracted.

The extracted *F* value corresponds to the following: "*Average lightness was higher in winners compared to both losers and males categorized as having no clear outcome in the first two rounds of the tournament (Table 2; Round 1 F~2,45~ = 6.88, P = 0.002)*"

-   **Our assessment**: Since the Lighter are the "Light Color", the sign of the final effect size should indeed be negative, which is. Nonetheless, the sample size should be 48 (i.e., 17+14+17) instead of 46 according to Table 2.

-   **Required action**: Change the sample size to 48, and recalculate *vi* to account for this change.

```{r}
metadata.new.updated[metadata.new.updated$Study=="Naretto et al 2023","Sample.Size"] <- 48

yi.tmp <- metadata.new.updated[metadata.new.updated$Study=="Naretto et al 2023","yi"]
Sample.Size.tmp <- metadata.new.updated[metadata.new.updated$Study=="Naretto et al 2023","Sample.Size"]

metadata.new.updated[metadata.new.updated$Study=="Naretto et al 2023","vi"] <- ((1-(yi.tmp^2))^2)/(Sample.Size.tmp-1)
```

When revisiting this study, we realized that there were additional effect sizes that could have been extracted corresponding to Rounds 2 and 3: "*...; Round 2 F~2,39~ = 5.752, P = 0.006; Round 3 F~2,37~ = 1.344, P = 0.273)*".

-   **Our assessment**: Those two effect sizes come from the same group of animals. In the full dataset there are several other papers where multiple effect sizes from the same group of animals were extracted. This sort of nonindependence (i.e., multiple estimates from the same group of animals) should be accounted for with a random effect (i.e., "Group ID"). This should be done for all such cases in the dataset.

-   **Required action**: To extract these effect sizes, we first confirm that indeed the direction should remain negative by checking Table 2, and then transforming those two *F* values as we did for the *F* value above.

```{r}
Naretto_and_Chiaraviglio_2023_extra_1 <- metadata.new %>% filter(Authors=="Naretto, Sergio; Chiaraviglio, Margarita")

Naretto_and_Chiaraviglio_2023_extra_2 <- metadata.new %>% filter(Authors=="Naretto, Sergio; Chiaraviglio, Margarita")

# emptying entry
Naretto_and_Chiaraviglio_2023_extra_1[,c(1:ncol(Naretto_and_Chiaraviglio_2023_extra_1))] <- NA

Naretto_and_Chiaraviglio_2023_extra_2[,c(1:ncol(Naretto_and_Chiaraviglio_2023_extra_2))] <- NA


# adding variables of interest from original sources
Naretto_and_Chiaraviglio_2023_extra_1[,c("Authors","Publication.Year","Species")] <- Naretto_and_Chiaraviglio_2023_subset[,c("Authors","Publication.Year","Species")]

Naretto_and_Chiaraviglio_2023_extra_2[,c("Authors","Publication.Year","Study","Species")] <- Naretto_and_Chiaraviglio_2023_subset[,c("Authors","Publication.Year","Study","Species")]

# Round 2 value
Naretto_and_Chiaraviglio_2023_extra_1[,"Study"] <- "Naretto et al 2023 - 2"
Naretto_and_Chiaraviglio_2023_extra_1[,"Sample.Size"] <- (9+24+9)
Naretto_and_Chiaraviglio_2023_extra_1[,"Stat.Test"] <- "F"
Naretto_and_Chiaraviglio_2023_extra_1[,"Test.Statistic"] <- 5.752
Naretto_and_Chiaraviglio_2023_extra_1[,"df1"] <- 2
Naretto_and_Chiaraviglio_2023_extra_1[,"df2"] <- 39

# caculating corresponding r
df1.tmp <- Naretto_and_Chiaraviglio_2023_extra_1[,"df1"]
df2.tmp <- Naretto_and_Chiaraviglio_2023_extra_1[,"df2"]
Test.Statistic.tmp <- Naretto_and_Chiaraviglio_2023_extra_1[,"Test.Statistic"]

Naretto_and_Chiaraviglio_2023_extra_1[,"yi"] <- sqrt((df1.tmp*Test.Statistic.tmp)/(df1.tmp*Test.Statistic.tmp+df2.tmp))

# adjusting the sign accordingly 
Naretto_and_Chiaraviglio_2023_extra_1[,"yi"] <- Naretto_and_Chiaraviglio_2023_extra_1[,"yi"]*(-1)

# calculating vi
yi.tmp <- Naretto_and_Chiaraviglio_2023_extra_1[,"yi"]
Sample.Size.tmp <- Naretto_and_Chiaraviglio_2023_extra_1[,"Sample.Size"]
Naretto_and_Chiaraviglio_2023_extra_1[,"vi"] <- ((1-(yi.tmp^2))^2)/(Sample.Size.tmp-1)

# Round 3 value
Naretto_and_Chiaraviglio_2023_extra_2[,"Study"] <- "Naretto et al 2023 - 3"
Naretto_and_Chiaraviglio_2023_extra_2[,"Sample.Size"] <- (5+30+5)
Naretto_and_Chiaraviglio_2023_extra_2[,"Stat.Test"] <- "F"
Naretto_and_Chiaraviglio_2023_extra_2[,"Test.Statistic"] <- 1.344
Naretto_and_Chiaraviglio_2023_extra_2[,"df1"] <- 2
Naretto_and_Chiaraviglio_2023_extra_2[,"df2"] <- 37

# calculating corresponding r
df1.tmp <- Naretto_and_Chiaraviglio_2023_extra_2[,"df1"]
df2.tmp <- Naretto_and_Chiaraviglio_2023_extra_2[,"df2"]
Test.Statistic.tmp <- Naretto_and_Chiaraviglio_2023_extra_2[,"Test.Statistic"]

Naretto_and_Chiaraviglio_2023_extra_2[,"yi"] <- sqrt((df1.tmp*Test.Statistic.tmp)/(df1.tmp*Test.Statistic.tmp+df2.tmp))

# adjusting the sign accordingly 
Naretto_and_Chiaraviglio_2023_extra_2[,"yi"] <- Naretto_and_Chiaraviglio_2023_extra_2[,"yi"]*(-1)

# calculating vi
yi.tmp <- Naretto_and_Chiaraviglio_2023_extra_2[,"yi"]
Sample.Size.tmp <- Naretto_and_Chiaraviglio_2023_extra_2[,"Sample.Size"]
Naretto_and_Chiaraviglio_2023_extra_2[,"vi"] <- ((1-(yi.tmp^2))^2)/(Sample.Size.tmp-1)

# finally, adding this entry to the new dataset
metadata.new.updated <- rbind(metadata.new.updated,
                              Naretto_and_Chiaraviglio_2023_extra_1,
                              Naretto_and_Chiaraviglio_2023_extra_2)
```

In addition, the study also provides three additional tests corresponding to differences in lightness before the trials: "*There were no significant differences in lightness before the beginning of each trial between categories (Table 2; Opponent A and Opponent B in Round 1: F ~1,46~ = 0.003, P = 0.955; W, NCO and L in Round 2: F~2,39~ = 1.604, P = 0.214; W, NCO and L in Round 3: F ~2,37~ = 0.661, P = 0.523).*".

-   **Our assessment**: From what is provided, we consider these set of three effect sizes alternative to the three already extracted, meaning that there is a reasonable argument for deciding whether to extract the effect sizes before or after the trial depending on the question at hand. Since the Ruckman et al. 2024 decided to extract the post-trial values, we will use that reasoning for not extracting these three additional effect sizes - note that adding these three additional effect sizes would, overall, further reduce the overall effect size.

-   **Required action**: None.

## Martin and Hengstebeck 1981

This study appears in the dataset as:

```{r}
Martin_and_Hengstebeck_1981_subset <- metadata.new %>% filter(Authors=="MARTIN, FD; HENGSTEBECK, MF") %>% select(all_of(columns.of.interest))

knitr::kable(Martin_and_Hengstebeck_1981_subset,format = "html")
```

<br>

For this species (i.e., `r unique(Martin_and_Hengstebeck_1981_subset$Species)`), Ruckman et al. (2024) classified *Less black eye* as the "Light Color" and *Darker eye* as the "Dark Color". Nine effect sizes were extracted.

The extracted *F* value corresponds to the following: "*Mean bout lengths of aggressive encounters were 10.3 s for dark-eyed fish, 7.2 s for intermediate fish, and 1.8 s for light-eyed fish (F~2,17~ = 3.80, P \< 0.005)*".

-   **Our assessment**: We think that the extracted *F* value is comparable to those extracted from other studies, and the results suggest that dark-eye fish spend more time on aggressive encounters than light-eyed fish. However, the sample size assigned (74) is not correct based on df2 (17). 74 seems to be the sets of observations performed not the number of individuals.

-   **Required action**: Change sample size to 17+2 = 19.

```{r}
metadata.new.updated[metadata.new.updated$Study=="Martin and Hengstebeck 1981 - 1","Sample.Size"] <- 17+2

yi.tmp <- metadata.new.updated[metadata.new.updated$Study=="Martin and Hengstebeck 1981 - 1","yi"]
Sample.Size.tmp <- metadata.new.updated[metadata.new.updated$Study=="Martin and Hengstebeck 1981 - 1","Sample.Size"]

metadata.new.updated[metadata.new.updated$Study=="Martin and Hengstebeck 1981 - 1","vi"] <- ((1-(yi.tmp^2))^2)/(Sample.Size.tmp-1)
```

The 8 extracted *X^2^* values corresponds to Table IV, where the provided values do not correspond to number of individuals but to number of encounters.

-   **Our assessment**: The study does not provide the number of individuals observed to generate the data presented in Table IV, not even an approximate number. The only information on sample sizes is: "*Litters selected for observations had a minimum number of five fish, and for Indiana fish the observed maximum was 21. Some of the Puerto Rico fish were removed on the first day after birth so that the maximum number in a tank was 11*", but the number of tanks is not reported. For all we can see, all the observations could come from an extremely low number of individuals (even 3, if one would go to the extreme).

-   **Required action**: We do not think the *X^2^* values can be reliably use for the meta-analysis as the sample size is unknown and the raw data not present, and therefore, we are excluding them from the dataset.

```{r}
#saving the useful entry
Martin_and_Hengstebeck_1981.tmp <- metadata.new.updated[metadata.new.updated$Study=="Martin and Hengstebeck 1981 - 1",]

#deleting the rest
metadata.new.updated <- metadata.new.updated[metadata.new.updated$Authors!="MARTIN, FD; HENGSTEBECK, MF",]

# adding the study back
metadata.new.updated <- rbind(metadata.new.updated,Martin_and_Hengstebeck_1981.tmp)
```

## Dijkstra et al 2009b

This study appears in the dataset as:

```{r}
Dijkstra_et_al_2009b_subset <- metadata.new %>% filter(Authors=="Dijkstra, PD; van Dijk, S; Groothuis, TGG; Pierotti, MER; Seehausen, O") %>% select(all_of(columns.of.interest))

knitr::kable(Dijkstra_et_al_2009b_subset,format = "html")
```

<br>

For this species (i.e., `r unique(Dijkstra_et_al_2009b_subset$Species)`), Ruckman et al. (2024) classified *Plain (orange)*as the "Light Color" and *Black* as the "Dark Color". Note that Ruckman et al. (2024) wrote that aggression between different morphs were said to be excluded "we therefore limit our data set to measure of aggression within color morphs". One effect size was extracted.

The extracted *X^2^* value corresponds to: "*The female morphs differed significantly in ranking (ranking mean+-SE: OB female 1.9 +/- 0.2; P female 2.6 +/-0.5; WB female 1.5 +/- 0.2, Friedman test, X^2^ = 9.50, df = 2, P = 0.009, n = 12)*"

-   **Our assessment**: The authors of the original study report: "*3 distinct female color morphs coexist, black-and-white blotched (WB), orange blotched (OB), and plain (P) color morphs. First, we investigated dominance relationships among female morphs using triadic and dyadic encounters in the laboratory*". We assume therefore assume that the three morphs are part of a continuum with the extremes being P (plain) and WB (black-and-white), and orange blotched (OB) being intermediate. As far as we can see everything seems correct with the data extraction from this study.

-   **Required action**: None.

## Yang et al 2018

This study appears in the dataset as:

```{r}
Yang_et_al_2018_subset <- metadata.new %>% filter(Authors=="Yang, Y; Dugas, MB; Sudekum, HJ; Murphy, SN; Richards-Zawacki, CL") %>% select(all_of(columns.of.interest))

knitr::kable(Yang_et_al_2018_subset,format = "html")
```

<br>

For this species (i.e., `r unique(Yang_et_al_2018_subset$Species)`), Ruckman et al. (2024) classified *Light red*, *Green* and *Red* as the "Light Color" and *Dark red*, *Red* and *Blue* as the "Dark Color", respectively. Note that Ruckman et al. (2024) wrote that aggression between different morphs were said to be excluded "we therefore limit our data set to measure of aggression within color morphs". Eight effect sizes were extracted.

The extracted *X^2^* values corresponds to Table S4-S7, where Likelihood Ratio (LR) X^2^ are presented: "*The four tables below are generalized linear models evaluating the influence of male color (red, intermediate and blue), model intruder color (red, blue) and their interaction term on the likelihood of a territorial male to track (Table S4), approach (Table S5), call (Table S6) and challenge (Table S7) in the two polymorphic populations. Perch height and conspecific interaction (y/n) were included as covariates*"

-   **Our assessment**: The direction of the provided *X^2^* values in Tables S4-S7 (as well as the general ones provided in Tables 2 and S3) is not provided in the original study. The authors of the original study only provide a direction of the effect for those that are statistically significant, e.g. "*When considering all territorial males, regardless of interaction with conspecifics during the trial, neither the main effects of male colour and intruder colour nor their interaction was a significant predictor of the probability of attack in the high-red polymorphic population (Table 2).*" or "*GLMs for the other four variables (likelihood to track, approach, call and challenge) are presented in Tables S4--S7. We did not detect any significant main effects or an interaction between male colour and model intruder colour in any of the models*". The only indication for Bluer males being more aggressive than Redder males come from the high-blue polymorphic population in Table S3, where the authors of the original study reported "*However, the likelihood of attack was positively correlated with PC2 (a hue indicator that increases with male 'blueness'; Table S1), suggesting that bluer males were more aggressive than redder males*". Table S3 shows PC1 (*X^2^* = 0.45, p-value = 0.505) and PC2 (*X^2^* = 6.49, p-value = 0.011), which are quantitative measures as opposed to the "by-eye male colour" categorizations presented in Tables 2 and S4-S7 (Table S10 shows similar results to Table S3 but for "*the subset of observations in which the focal male did not interact with a conspecific*"). According to the authors of the original study, "*PC1 captures the brightness (but much higher green and blue loading) of the male dorsum; PC2 captures hue, or how blue the male was along the red-blue spectrum*". Summarizing, from the reported results, it is not possible to know the direction of any reported *X^2^* value other than PC2 in Table S3 (*X^2^* = 6.49, p-value = 0.011) and the corresponding one in Tables S9 and S10, which present a subset of the same data used in Tables 2 and S3. Thus, without additional information, we cannot assume all those *X^2^* values are positive.

-   **Required action**: Exclude the study. For 7 out of 8 *X^2^* we do not know the direction of the effect. The only *X^2^* value for which we know the direction (PC2: *X^2^* = 6.49, p-value = 0.011, Table S3) is the one corresponding to PC2 in Table S3 - however the corresponding PC1 (which reflects male brightness) is statistically nonsignificant and we do not know in which direction. Thus we think that extracting the only *X^2^* value for which we know the direction would lead to a biased representation of the findings of the study. Last, all *X^2^* values provided come from Binomial GLMs rather than *X^2^* tests, adding additional complexity to their transformation into an effect size.

```{r}
#deleting the study
metadata.new.updated <- metadata.new.updated[metadata.new.updated$Authors!="Yang, Y; Dugas, MB; Sudekum, HJ; Murphy, SN; Richards-Zawacki, CL",]
```

## Rose and Soole 2020

This study appears in the dataset as:

```{r}
Rose_and_Soole_2020_subset <- metadata.new %>% filter(Authors=="Rose, P; Soole, L") %>% select(all_of(columns.of.interest))

knitr::kable(Rose_and_Soole_2020_subset,format = "html")
```

<br>

For this species (i.e., `r unique(Rose_and_Soole_2020_subset$Species)`), Ruckman et al. (2024) classified *White* as the "Light Color" and *Pink* as the "Dark Color". One effect size was extracted.

The extracted *F* value corresponds to: "*Differences in time spent on aggression and plumage colour score are significant between birds (F~4,40~ = 6.45; r^2^ = 33%; p = .0004).*"

-   **Our assessment**: In this study, plumage colour is scored in four categories: 1 being white, and 2 being pink, and from the results shown in Figure 7, from which the *F* value was extracted, it is clear that, as reported in the original study: "*Figure 7 shows that the brightest flamingos are least likely to be seen foraging and being aggressive regardless of the type of foraging location. Birds with a colour score of 3 were most often seen being aggressive; birds with a colour score of 4 had the lowest foraging occurrences*". Thus, extracting the *F* value, which corresponds to an omnibus test on all four categories would be rather misleading. Instead, the most straightforward way of extracting this result would have been directly from the figure. However, sample sizes for each category are missing. Thus, the second best choice here would be to extract the *t* value, which despite seemingly coming from a GLM, explicitly shows: "*Birds with a brighter plumage are more likely to be aggressive during foraging than paler birds (estimate = 10.23; SE = 4.88; t value = 2.09; p = .04).*"

-   **Required action**: To substitute the extracted *F* value by the *t* value, delete the extracted *r* value, which corresponds to the *R^2^* value of the *F* value, and recalculate the corresponding *r* and *Vr* values using `escalc()` as done for the other *t* values.

```{r}
# making changes 
metadata.new.updated[metadata.new.updated$Study=="Rose and Soole 2020","Stat.Test"] <- "t"
metadata.new.updated[metadata.new.updated$Study=="Rose and Soole 2020","Test.Statistic"] <- 2.09 # no sign change needed
metadata.new.updated[metadata.new.updated$Study=="Rose and Soole 2020","df1"] <- NA
metadata.new.updated[metadata.new.updated$Study=="Rose and Soole 2020","df2"] <- NA
metadata.new.updated[metadata.new.updated$Study=="Rose and Soole 2020","p.value"] <- 0.04
metadata.new.updated[metadata.new.updated$Study=="Rose and Soole 2020","r"] <- NA

# adding the corresponding yi and vi values
metadata.new.updated[metadata.new.updated$Study=="Rose and Soole 2020","yi"] <- escalc(measure = "COR",
                                                                                       ti = 2.09,
                                                                                       ni = 45)[1]
metadata.new.updated[metadata.new.updated$Study=="Rose and Soole 2020","vi"] <- escalc(measure = "COR",
                                                                                       ti = 2.09,
                                                                                       ni = 45)[2]
```

## Podberscek and Serpell 1996

This study appears in the dataset as:

```{r}
Podberscek_and_Serpell_1996_subset <- metadata.new %>% filter(Authors=="Podberscek, AL; Serpell, JA") %>% select(all_of(columns.of.interest))

knitr::kable(Podberscek_and_Serpell_1996_subset,format = "html")
```

<br>

For this species (i.e., `r unique(Podberscek_and_Serpell_1996_subset$Species)`), Ruckman et al. (2024) classified *Black* as the "Light Color" and *Red/golden* as the "Dark Color". Ten effect sizes were extracted (83% of all mammal ones, 10/12).

Results are shown in: "*Within the solid colour group, red/goldens were compared with blacks. Here it was found that red/goldens were significantly more likely to be aggressive in a number of situations. These included, Al (towards strange dogs; Mann-Whitney U test, Z = 2.582, P \< 0.01), A4 (towards persons approaching owner away from home; Z = 2.774, P \< 0.011, A5 (towards children in the household; Z= 3.365, P \< 0.001), A7 (when owner gives attention to other person or animal; Z = 3.336, P \< 0.001), A8 (toward owner or member of owner's family; Z= 4.988, P \< 0.001), A9 (when disciplined; Z= 4.524, P \< 0.001)\>, A10 (when reached for or handled; Z= 3.161, P \< 0.011, All (when in restricted spaces; Z = 2.4, P \< 0.05\>, Al2 (at meal times/ defending food; Z = 3.492, P \< 0.001)), Al3 (sudden and without apparent reason; Z= 3.643, P \< 0.001).*"

-   **Our assessment**: Ruckman et al. 2024 established the following criterium: "*We defined aggression as any variable that measured antagonistic behaviors (e.g., biting or chasing) toward a conspecific (of same sex, color class, and age class) or mirror image.*" Of the 13 questions asked of the dogs' owners in Podberscek and Serpell 1996, only two (A1 and A6) refer to aggression towards conspecifics (A1 and A6), see table 1 of the original paper. Hence, only these two should be considered. Of these 2, only 1 (A1) is significant and therefore reported, as only statistically significant findings were reported. Only extracting significant results would bias the results, and therefore, even this single effect size should be excluded. Moreover, the methodology used (dog owner surveys) is not at all comparable with the rest of the studies where aggression was measured directly, and therefore, we think that the study should have been excluded a priori in any case.

-   **Required action**: Exclude this study.

```{r}
metadata.new.updated <- metadata.new.updated[metadata.new.updated$Authors!="Podberscek, AL; Serpell, JA",]
```

## Karlsson et al. 2011

This study appears in the dataset as:

```{r}
Karlsson_et_al_2011_subset <- metadata.new %>% filter(Authors=="Karlsson, AC; Mormede, P; Kerje, S; Jensen, P") %>% select(all_of(columns.of.interest))

knitr::kable(Karlsson_et_al_2011_subset,format = "html")
```

<br>

For this species (i.e., `r unique(Karlsson_et_al_2011_subset$Species)`), Ruckman et al. (2024) classified *White* as the "Light Color" and *Wild type (red)* as the "Dark Color". Note that Ruckman et al. (2024) wrote that aggression between different morphs were said to be excluded "we therefore limit our data set to measure of aggression within color morphs". Four effect sizes were extracted.

Results are shown in Table 2.

-   **Our assessment**: All values were extracted correctly.

-   **Required action**: None.

## Seaver and Hurd 2017

This study appears in the dataset as:

```{r}
Seaver_and_Hurd_2017_subset <- metadata.new %>% filter(Authors=="Seaver, CMS; Hurd, PL") %>% select(all_of(columns.of.interest))

knitr::kable(Seaver_and_Hurd_2017_subset,format = "html")
```

<br>

For this species (i.e., `r unique(Seaver_and_Hurd_2017_subset$Species)`), Ruckman et al. (2024) classified *Yellow* as the "Light Color" and *Red* as the "Dark Color". Note that Ruckman et al. (2024) wrote that aggression between different morphs were said to be excluded "we therefore limit our data set to measure of aggression within color morphs". Four effect sizes were extracted.

The effects sizes were seemingly extracted from Figure 2.

-   **Our assessment**: All values were extracted correctly. However, there seem to be an additional effect size that could have been extracted: "*There was no significant difference between females, yellow males and red males in the proportion that showed aggression to their mirror image (X^2^~2~ = 3.20, p = 0.20; Table 1)*". The corresponding 2x2 contingency table for that result would be:

```{r}
#create table
table1.Seaver <- matrix(c(19,51,4,11), ncol=2, byrow=TRUE)
rownames(table1.Seaver) <- c("yellow","red")
colnames(table1.Seaver) <- c("nonaggressor","aggressor")
table1.Seaver <- as.table(table1.Seaver)
table1.Seaver
```

From which we can calculate the corresponding X^2^ value as:

```{r}
#chisq.test(table1.Seaver)
chisq.test(table1.Seaver,correct=F)
```

<br>

-   **Required action**: To add this additional effect size by transforming the X^2^ value to an *r* value following the procedures used for the other studies.

```{r}
Seaver_and_Hurd_2017_extra_1 <- metadata.new %>% filter(Study=="Seaver and Hurd 2017 - 1")

# emptying entry
Seaver_and_Hurd_2017_extra_1[,c(1:ncol(Seaver_and_Hurd_2017_extra_1))] <- NA


# adding variables of interest from original sources
Seaver_and_Hurd_2017_extra_1[,c("Authors","Publication.Year","Species")] <- Seaver_and_Hurd_2017_subset[1,c("Authors","Publication.Year","Species")]

Seaver_and_Hurd_2017_extra_1[,"Study"] <- "Seaver and Hurd 2017 - 5"
Seaver_and_Hurd_2017_extra_1[,"Sample.Size"] <- sum(table1.Seaver)
Seaver_and_Hurd_2017_extra_1[,"Stat.Test"] <- "X2"
Seaver_and_Hurd_2017_extra_1[,"Test.Statistic"] <- chisq.test(table1.Seaver,correct=F)$statistic[[1]]
Seaver_and_Hurd_2017_extra_1[,"df1"] <- chisq.test(table1.Seaver,correct=F)$parameter[[1]]
Seaver_and_Hurd_2017_extra_1[,"p.value"] <- chisq.test(table1.Seaver,correct=F)$p.value[[1]]

# caculating corresponding r
Seaver_and_Hurd_2017_extra_1[,"yi"] <- sqrt((chisq.test(table1.Seaver,correct=F)$statistic[[1]])/
                                              (sum(table1.Seaver)*(nrow(table1.Seaver)-1)))

# calculating vi
Seaver_and_Hurd_2017_extra_1[,"vi"] <- ((1 - (Seaver_and_Hurd_2017_extra_1[,"yi"] ^ 2)) ^ 2)/(sum(table1.Seaver) - 1)

# finally, adding this entry to the new dataset
metadata.new.updated <- rbind(metadata.new.updated,
                              Seaver_and_Hurd_2017_extra_1)


```

## Zinzow-Kramer et al. 2015

This study appears in the dataset as:

```{r}
Zinzow_Kramer_et_al_2015_subset <- metadata.new %>% filter(Authors=="Zinzow-Kramer, WM; Horton, BM; McKee, CD; Michaud, JM; Tharp, GK; Thomas, JW; Tuttle, EM; Yi, S; Maney, DL") %>% select(all_of(columns.of.interest))

knitr::kable(Zinzow_Kramer_et_al_2015_subset,format = "html")
```

<br>

For this species (i.e., `r unique(Zinzow_Kramer_et_al_2015_subset$Species)`), Ruckman et al. (2024) classified *White (WS)* as the "Light Color" and *Tan (TS)* as the "Dark Color". Eight effect sizes were extracted.

The effects sizes were extracted from Figure 1.

-   **Our assessment**: All values extracted are correct, but there are two effect sizes for which the sign have been flipped. Those two correspond to latency to approach (the time from start of playback until the resident male arrived: the longer, the more scared), and distance of closest approach to the decoy (the further, the more scared). In addition, the extracted values correspond to medians rather than means, which should have probably accounted for since medians can be rather far from means when data is skewed (for more on this, see https://training.cochrane.org/handbook/current/chapter-06#section-6-5-2). We ignore this last issue as likely inconsequential.

-   **Required action**: To assign a negative sign to effect sizes corresponding to latency to approach and instance of closest approach to the decoy.

```{r}
# adjusting the sign accordingly 
metadata.new.updated[metadata.new.updated$Study=="Zinzow-Kramer et al 2015 - 7","yi"] <- metadata.new.updated[metadata.new.updated$Study=="Zinzow-Kramer et al 2015 - 7","yi"]*(-1)
metadata.new.updated[metadata.new.updated$Study=="Zinzow-Kramer et al 2015 - 8","yi"] <- metadata.new.updated[metadata.new.updated$Study=="Zinzow-Kramer et al 2015 - 8","yi"]*(-1)
```

# Describing differences between original and updated dataset

After accounting for the issues found in 8 out 11 studies (`r round(100*7/11,0)`%) that were reassessed and that correspond to `r round(100*11/length(unique(metadata.new$Authors)),0)`% of all studies included in Ruckman et al. (2024), the **new and updated dataset** contains `r nrow(metadata.new.updated)` effect sizes extracted from `r length(unique(metadata.new.updated$Authors))` studies and covering `r length(unique(metadata.new.updated$Species))` species\*, whereas the **original dataset** contained `r nrow(metadata.new)` effect sizes extracted from `r length(unique(metadata.new$Authors))` studies and covering `r length(unique(metadata.new$Species))` species\*.

\* Note that the final number of species for the analysis is 54 because we renamed *Haplochromis omnicaeruleus* as *Haplochromis paludinosus* following the updated taxonomic information.

Our reassessment allowed us to reduce (but likely not eliminate) the consequences of the excess of positive values found in the original dataset, which, based on our reassessment we believe to be largely caused by an incorrect management of effect size direction (more below). Indeed, whereas the percentage of positive values for each effect size origin for the **original dataset** looked like:

```{r}
################################################################################
# Exploring effect size type disagreements
################################################################################

# calculate percentage of positive values for each type of effect size for
# the original
effect.size.positive.perc.original <- metadata.new %>% 
  group_by(Stat.Test) %>% 
  mutate(Stat.Test = factor(Stat.Test, 
                            levels = c("r","mean","t","F","X2"))) %>%
  mutate(Stat.Test = recode(Stat.Test, r = "Pearson's r",
                            mean = "mean, SD, N",
                            t = "t value",
                            F = "F value",
                            X2 = "X2 value")) %>% 
  summarise(Percentage = round(100*table(yi<0)[1]/n(),1))# %>% 

knitr::kable(effect.size.positive.perc.original,format = "html") # output format specification is optional

```

The corresponding percentages for the **new and updated** dataset looked like:

```{r}
# and the updated database
effect.size.positive.perc.updated <- metadata.new.updated %>%
  group_by(Stat.Test) %>%
  mutate(Stat.Test = factor(Stat.Test,
                            levels = c("r","mean","t","F","X2"))) %>%
  mutate(Stat.Test = recode(Stat.Test, r = "Pearson's r",
                            mean = "mean, SD, N",
                            t = "t value",
                            F = "F value",
                            X2 = "X2 value")) %>%
  summarise(Percentage = round(100*table(yi<0)[1]/n(),1))# %>% 

knitr::kable(effect.size.positive.perc.updated,format = "html") # output format specification is optional
```

Here are the corresponding figures for the **original dataset**:

```{r}
# generating the data subset
metadata.original.yi <- metadata.new %>%
  select(c(yi,vi,Stat.Test)) %>%
  mutate(Stat.Test = factor(Stat.Test,
                            levels = c("r","mean","t","F","X2"))) %>%
  mutate(Stat.Test = recode(Stat.Test, r = "Pearson's r",
                            mean = "mean, SD, N",
                            t = "t value",
                            F = "F value",
                            X2 = "X2 value"))

# generating label for annotation
effect.size.positive.perc.original$label.perc <- paste0(round(effect.size.positive.perc.original$Percentage,0),
                                                        "%\npositive")


# effect size magnitude # more at: https://indrajeetpatil.github.io/ggstatsplot/reference/ggbetweenstats.html
set.seed(77)
yi.plot.original <- ggbetweenstats(
  data  = metadata.original.yi,
  x     = Stat.Test,
  y     = yi,
  point.args = list(position = ggplot2::position_jitterdodge(dodge.width = 0.6),
                    alpha = 0.4,
                    size = 1/sqrt(metadata.original.yi$vi)-min(1/sqrt(metadata.original.yi$vi))+0.1,
                    stroke = 0, na.rm = TRUE),
  #point.args = list(size = 1),
  type = "parametric",
  pairwise.display = "none",
  #p.adjust.method = "none", # if no multiple correction used, differences are everywhere
  #ggsignif.args = list(textsize = 3, tip_length = 0.02, na.rm = TRUE), # if pairwise.display on, change size
  bf.message = F,
  effsize.type = "eta", # which corresponds to the partial eta squared we are using to transform F-to-r
  #results.subtitle = F, # to remove statistical results from the top of the plot
  centrality.label.args = list(size = 3, nudge_x = 0.4,
                               segment.linetype = 3,
                               min.segment.length = 0),
  xlab = "\nEffect size origin\n",
  ylab = "\nEffect size\n(r and rbis)",
  title = "\nDoes effect size magnitude differ considerably depending on their origin?"
) +
  # modifying text size
  theme(axis.text=element_text(size=10),
        axis.title=element_text(size=11,face="bold"),
        plot.title = element_text(size=12)) +
  # adding the percentage of positive effect sizes for each type
  annotate("text",
           x = seq(0.65,4.65,1),
           y = 1.1,
           label = effect.size.positive.perc.original$label.perc) +
  # adding grey area to better signal postive vs negative values
  annotate("rect", xmin = 0, xmax = 6, ymin = -1, ymax = 0,
           alpha = .1)

yi.plot.original
```

and the **new and updated dataset**:

```{r}
# generating the data subset
metadata.updated.yi <- metadata.new.updated %>%
  select(c(yi,vi,Stat.Test)) %>%
  mutate(Stat.Test = factor(Stat.Test,
                            levels = c("r","mean","t","F","X2"))) %>%
  mutate(Stat.Test = recode(Stat.Test, r = "Pearson's r",
                            mean = "mean, SD, N",
                            t = "t value",
                            F = "F value",
                            X2 = "X2 value"))

# generating label for annotation
effect.size.positive.perc.updated$label.perc <- paste0(round(effect.size.positive.perc.updated$Percentage,0),
                                                        "%\npositive")


# effect size magnitude # more at: https://indrajeetpatil.github.io/ggstatsplot/reference/ggbetweenstats.html
set.seed(77)
yi.plot.updated <- ggbetweenstats(
  data  = metadata.updated.yi,
  x     = Stat.Test,
  y     = yi,
  point.args = list(position = ggplot2::position_jitterdodge(dodge.width = 0.6),
                    alpha = 0.4,
                    size = 1/sqrt(metadata.updated.yi$vi)-min(1/sqrt(metadata.updated.yi$vi))+0.1,
                    stroke = 0, na.rm = TRUE),
  #point.args = list(size = 1),
  type = "parametric",
  pairwise.display = "none",
  #p.adjust.method = "none", # if no multiple correction used, differences are everywhere
  #ggsignif.args = list(textsize = 3, tip_length = 0.02, na.rm = TRUE), # if pairwise.display on, change size
  bf.message = F,
  effsize.type = "eta", # which corresponds to the partial eta squared we are using to transform F-to-r
  #results.subtitle = F, # to remove statistical results from the top of the plot
  centrality.label.args = list(size = 3, nudge_x = 0.4,
                               segment.linetype = 3,
                               min.segment.length = 0),
  xlab = "\nEffect size origin\n",
  ylab = "\nEffect size\n(r and rbis)",
  title = "\nDoes effect size magnitude differ considerably depending on their origin?"
) +
  # modifying text size
  theme(axis.text=element_text(size=10),
        axis.title=element_text(size=11,face="bold"),
        plot.title = element_text(size=12)) +
  # adding the percentage of positive effect sizes for each type
  annotate("text",
           x = seq(0.65,4.65,1),
           y = 1.1,
           label = effect.size.positive.perc.updated$label.perc) +
  # adding grey area to better signal postive vs negative values
  annotate("rect", xmin = 0, xmax = 6, ymin = -1, ymax = 0,
           alpha = .1)

yi.plot.updated
```

Based on our exploration of `r round(100*11/length(unique(metadata.new$Authors)),0)`% of all studies included in Ruckman et al. (2024), the excess of positive values found is likely due to an incorrect assignment of effect size direction in the original dataset due to: (1) not adjusting the direction of effect size of traits for which larger means less aggressive (e.g., latency to approach), (2) assigning a positive sign to directionless inferential statistics such as *F* and *X^2^* values, and (3) an unexpected lower likelihood of negative effect sizes.

# Conclusions

Based on our reassessment of `r round(100*11/length(unique(metadata.new$Authors)),0)`% of all studies included in Ruckman et al. (2024) we cannot guarantee the reliability of the dataset. That is, despite that we have fixed the issues found in 73% of all reassessed studies, there is strong evidence suggesting that those (and possibly other) issues will be present for a substantial percentage of the remaining `r 100-round(100*11/length(unique(metadata.new$Authors)),0)`% of the studies that we did not reassessed. Thus, our re-analyses should be interpret with extreme caution as there is evidence to expect that their results will still exaggerate the *true* association between aggression and coloration.

The code below saves the *new and updated dataset* for the corresponding analyses.

```{r}
# saving dataset
write.csv(metadata.new.updated, "../data/new/meta_complete_data2_new_and_updated.csv")

```

### R session info

```{r}
sessionInfo()
```
```
